# Identification of Rice Genes Associated With Enhanced Cold Tolerance by Comparative Transcriptome Analysis With Two Transgenic Rice Plants Overexpressing *DaCBF4* or *DaCBF7*, Isolated From Antarctic Flowering Plant *Deschampsia antarctica*

**DOI:** 10.3389/fpls.2018.00601

**Published:** 2018-05-03

**Authors:** Mi Young Byun, Li Hua Cui, Jungeun Lee, Hyun Park, Andosung Lee, Woo Taek Kim, Hyoungseok Lee

**Affiliations:** ^1^Unit of Polar Genomics, Korea Polar Research Institute, Incheon, South Korea; ^2^Department of Systems Biology, Yonsei University, Seoul, South Korea; ^3^Polar Science, University of Science & Technology, Daejeon, South Korea

**Keywords:** C-repeat/DRE binding factor, cold tolerance, *Deschampsia antarctica*, RNA-seq, transgenic plant, rice

## Abstract

Few plant species can survive in Antarctica, the harshest environment for living organisms. *Deschampsia antarctica* is the only natural grass species to have adapted to and colonized the maritime Antarctic. To investigate the molecular mechanism of the Antarctic adaptation of this plant, we identified and characterized *D. antarctica C-repeat binding factor 4* (*DaCBF4*), which belongs to monocot CBF group IV. The transcript level of *DaCBF4* in *D. antarctica* was markedly increased by cold and dehydration stress. To assess the roles of *DaCBF4* in plants, we generated a *DaCBF4*-overexpressing transgenic rice plant (*Ubi:DaCBF4*) and analyzed its abiotic stress response phenotype. *Ubi:DaCBF4* displayed enhanced tolerance to cold stress without growth retardation under any condition compared to wild-type plants. Because the cold-specific phenotype of *Ubi:DaCBF4* was similar to that of *Ubi:DaCBF7* (Byun et al., 2015), we screened for the genes responsible for the improved cold tolerance in rice by selecting differentially regulated genes in both transgenic rice lines. By comparative transcriptome analysis using RNA-seq, we identified 9 and 15 genes under normal and cold-stress conditions, respectively, as putative downstream targets of the two *D. antarctica* CBFs. Overall, our results suggest that Antarctic hairgrass *DaCBF4* mediates the cold-stress response of transgenic rice plants by adjusting the expression levels of a set of stress-responsive genes in transgenic rice plants. Moreover, selected downstream target genes will be useful for genetic engineering to enhance the cold tolerance of cereal plants, including rice.

## Introduction

Plants are sessile organisms and are constantly exposed to adverse environmental conditions, to which they have adapted (Lata and Prasad, 2011; Claeys and Inzé, 2013). The maritime Antarctic climate has low temperatures, high soil salinity, and a water deficit, which hamper the survival of angiosperms. *Deschampsia antarctica* Desv. (Poaceae) is one of two flowering plants that naturally inhabit Antarctica (Alberdi et al., 2002). This species has developed various adaptive mechanisms to survive in the Antarctic.

The adaptations that enable *D. antarctica* to survive in the harsh environment of the maritime Antarctic include changes in the leaf anatomy and the physiology of the photosynthetic apparatus (Giełwanowska et al., 2005; Sáez et al., 2017). Increased levels of nonstructural carbohydrates, apoplastic antifreeze proteins, and ice recrystallization inhibition proteins (biochemical cryoprotectants) reportedly enable plants to flourish under Antarctic conditions (Bravo and Griffith, 2005; John et al., 2009; Pastorczyk et al., 2014). In addition, transcriptome analysis of *D. antarctica* under abiotic stress demonstrated changes in the expression levels of stress-responsive genes (Lee et al., 2013a). However, the signaling pathways that mediate activation of the expression of the stress-related genes responsible for the abiotic stress tolerance of this plant are unclear.

In *Arabidopsis*, C-repeat binding factor (CBF)/dehydration-responsive element-binding protein (DREB) is the major transcription factor responsible for induction of cold tolerance (Thomashow, 2010). Also, *CBF* overexpression in monocot plants resulted in enhanced tolerance to diverse abiotic stresses including cold and freezing. When overexpressed in barley, *TaDREB3, TaCBF14*, and *TaCBF15* from wheat and *HvCBF2a* from barley resulted in enhanced frost tolerance by increasing the transcript levels of downstream target genes such as *COR14b* and *DHN5* (Kovalchuk et al., 2013; Soltész et al., 2013; Jeknić et al., 2014). Moreover, *TaCBF3* overexpression enhanced tolerance to frost and cold in wheat and barley (Morran et al., 2011). Expression of cotton *GhDREB* in barley also resulted in enhanced cold tolerance (Gao et al., 2009). Indeed, overexpression of the *CBF* genes from rice (*OsDREB1*), barley (*HvCBF4*), and maize (*ZmCBF3*) resulted in enhanced cold tolerance in rice (Ito et al., 2006; Oh et al., 2007; Xu et al., 2011).

Followed by the discovery of AtCBF1 as the major transcriptional regulator responsible for inducing the expression of downstream *COR* genes in *Arabidopsis* (Jaglo-Ottosen et al., 1998), the CBF proteins of monocot cereal plants have been discovered genome-wide (Stockinger, 2009; Tondelli et al., 2011). Moreover, Badawi et al. (2007) divided monocot CBF proteins into groups I–IV, and group V was recently identified (Byun et al., 2015).

However, few studies have aimed to identify the downstream target genes responsible for the cold tolerance in cereal plants. Overexpression of the rice *CBF* homolog *OsDREB1A*, have shown enhanced tolerance of transgenic rice to drought, high-salt, and low-temperature stresses. A microarray analysis of 21,500 rice genes together with northern blot analysis confirmed that 12 genes, including dehydrins and a protease inhibitor, were upregulated in *OsDREB1*-overexpressing rice plants (Ito et al., 2006). Rice plants continuously overexpressing *HvCBF4* from barley showed improved tolerance to cold, drought, and high-salt stresses. A microarray analysis revealed that the transcript levels of 15 genes, including Bowman Birk trypsin inhibitor 1 (*BBTI1*), were upregulated in *HvCBF4* overexpressing rice plants under normal condition (Oh et al., 2007).

In previous work, we generated transgenic rice plants overexpressing *DaCBF7*, a *CBF* gene from *D. antarctica*, and analyzed the expression levels of downstream genes. *DaCBF7* overexpression resulted in enhanced cold tolerance compared to wild-type plants (Byun et al., 2015). In this study, we found that the *D. antarctica CBF4* gene (*DaCBF4*) encodes a homolog of cereal CBF group IV. The expression of this gene was induced in *D. antarctica* plants under cold and drought stress. Overexpression of *DaCBF4* in rice resulted in a cold-specific phenotype similar to that of the *DaCBF7*-overexpressing plants. Thus, we identified the genes responsible for increasing the cold tolerance of rice by screening differentially regulated genes common to *DaCBF7*- and *DaCBF4*-overexpressing rice plants. Based on these results, we suggest that the Antarctic hairgrass *DaCBF4* gene plays a crucial role in the cold tolerance in transgenic rice plants. Selected downstream target genes common to *Ubi:DaCBF4* and *Ubi:DaCBF7* will be useful for genetic engineering to enhance the cold-stress tolerance of cereal plants, including rice.

## Materials and methods

### Phylogenetic analysis

The amino acid sequences of DaCBF4 and other CBF/DREB homologs from monocot crops were retrieved from the GenBank database and proofread. All downstream analyses were performed using the MEGA7 software (Kumar et al., 2016). Phylogenetic trees were constructed from the data sets by the neighbor-joining method based on the JTT matrix-based model. All positions with <95% site coverage were eliminated. Fewer than 5% alignment gaps, missing data, and ambiguous bases were allowed at any position. Supports for internal branches were tested by bootstrap analyses of 1,000 replications. The accession numbers of CBF homologs from five monocot species—Antarctic hairgrass (*D. antarctica*), barley (*Hordeum vulgare*), rice (*Oryza sativa*), hexaploid wheat (*Triticum aestivum*), and einkorn wheat (*Triticum monococcum*)—are presented in Supplementary Table 1.

### Subcellular localization experiment

The synthetic green fluorescent protein (*sGFP)-*coding region was fused in-frame to the 3′ end of the full-length *DaCBF4-*coding region and inserted into the pEarleyGate 100 binary vector. The vector was transformed into *Agrobacterium tumefaciens* strain GV3101 by electroporation. Tobacco (*Nicotiana benthamiana*) leaves were co-infiltrated using *A. tumefaciens* that contained the *35S:DaCBF4-sGFP* or *35S:sGFP* constructs. A *35S:*nuclear localizing signal-monomeric red fluorescent protein (*35S:NLS-mRFP*) construct was used as a control nuclear protein. Two days after infection, protoplasts were extracted from the tobacco leaves and visualized by fluorescence microscopy (BX51, Olympus).

### Plant materials and stress conditions

*Deschampsia antarctica* was collected near the King Sejong Antarctic Station (62°14′29″S; 58°44′18″W) on the Barton Peninsula of King George Island in January 2007. The plants were cultured *in vitro* in tissue culture medium [Murashige and Skoog (MS) medium; 2% sucrose and 0.8% phytoagar (pH 5.7)] under a 16-h light/8-h dark photoperiod with a light intensity of 150 μmol m^−2^ s^−1^ at 15°C. For cold-stress conditions, plants grown at 15°C were transferred to a climate chamber at 4°C for the indicated periods. For drought-stress conditions, plants were transferred to filter paper and dried at 15°C. For high-salt conditions, plants were transferred to MS medium supplemented with 150 mM NaCl and incubated at 15°C. RNA was extracted from leaves and analyzed at various times after the imposition of stress. All the samplings for expression analysis was conducted at the same time to avoid variation by circadian rhythm.

### Total RNA extraction and reverse transcription-quantitative polymerase chain reaction

Total RNA was isolated from leaves of five *D. antarctica* plants with four tillers each (longest tiller, 5 cm) using an RNeasy Plant Mini Kit in conjunction with the RNase free DNase set (Qiagen) according to the manufacturer's instructions. The quantity and quality of total RNA were determined by spectroscopic measurements at 230, 260, and 280 nm using an ND-1000 spectrophotometer (NanoDrop Technologies, Wilmington, DE), and RNA integrity was checked by electrophoresis in a 2% agarose gel. First-strand cDNA was synthesized from 2 μg of total RNA using Superscript III (Invitrogen). Real-time reverse transcription-quantitative polymerase chain reaction (RT-qPCR) analysis was performed in 20-μL reaction mixtures that included 1 μL of a 1:15 diluted cDNA template, 2 μM of each primer, and 10 μL of QuantiFast SYBR Green PCR kit (Qiagen). Amplified signals were monitored continuously using the Mx3000P qPCR System (Stratagene). The amplification protocol was as follows: 5 min of denaturation and enzyme activation at 95°C; followed by 40 cycles at 95°C for 5 s, 58°C for 20 s, and 72°C for 15 s. The *DaEF1a* gene was used as an internal control (Lee et al., 2010). The DNA sequences of primers used for PCR amplification are listed in Supplementary Table 2.

### Generation of *DaCBF4*-overexpressing transgenic rice plants

To generate *Ubi:DaCBF4* transgenic rice plants, the 651-bp *DaCBF4*-coding region was inserted into the pGA2897 vector. The binary vector was transformed into *A. tumefaciens* strain LBA4404 by electroporation and used for rice transformation as described previously (Byun and Kim, 2014). Callus tissue was induced from wild-type rice (*O. sativa* L. japonica variety “Dong-Jin”) seeds, co-cultivated with *A. tumefaciens*, and selected on callus induction medium containing antibiotics (40 mg/L hygromycin B and 250 mg/L carbenicillin). Selected callus tissue was transferred to a regeneration medium. Transgenic T0 plants were transplanted to soil in a greenhouse and T2 plants were used for phenotypic analysis.

### RT-PCR and DNA gel-blot analysis

Total RNA was isolated from mature leaves of wild-type and *Ubi:DaCBF4* transgenic rice plants using TRIzol reagent [38% equilibrated phenol, pH 4.3, 1 M guanidine thiocyanate, 1 M ammonium thiocyanate, 0.3 M sodium acetate, pH 5.2, and 5% glycerol (v/v)]. First-strand cDNA was synthesized from 2 μg of total RNA using oligo(dT) primers and TOPscript Reverse Transcriptase (Enzynomics). To compare the *DaCBF4* expression levels of the transgenic lines, *DaCBF4* was amplified using a gene-specific primer set (Supplementary Table 2). To distinguish the independent lines of *Ubi:DaCBF4* transgenic plants, genomic Southern blotting was performed. Total genomic DNA was isolated from mature rice leaves using cetyltrimethylammonium bromide (CTAB) solution, digested with *Bam*HI, and separated in a 0.8% agarose gel. The gel was treated sequentially with depurifying, denaturing, and neutralizing solutions, and the DNA was transferred to Hybond-N nylon membranes. The blot was hybridized with a ^32^P-labeled hygromycin B phosphotransferase (hph) probe under high-stringency conditions.

### Rice plant materials and stress conditions

Dry rice seeds were sterilized with 0.4% NaClO solution for 30 min and washed several times with sterilized water. Seeds of wild-type and *Ubi:DaCBF4* transgenic rice plants were germinated on half-strength MS medium containing vitamins (Duchefa Biochemie), 3% sucrose, and 0.7% phytoagar. Seedlings were grown for 2 weeks at 28°C under a 16-h light/8-h dark photoperiod and then transplanted to soil in a greenhouse as described by Byun et al. (2017). To investigate the effect of *DaCBF4* on stress tolerance, 6-week-old plants grown in the growth room were subjected to various abiotic stresses, observed, and photographed. For cold-stress conditions, wild-type and *DaCBF4-*overexpressing plants (independent lines #1 and #2) grown at 28°C were transferred to 4°C for 5 days, and then returned to 28°C.

### RNA-sequencing

RNA-sequencing (RNA-seq) was carried out using total RNA from 6-week-old wild-type and *DaCBF4-*overexpressing line #1 plants under normal and cold-stress conditions (1 or 6 days after transfer to 4°C). Total RNAs were extracted from leaves of each genotype and each treatment using TRIzol reagent, treated with DNase I to remove contaminant genomic DNA, and purified using the RNeasy Mini kit (Qiagen) following the manufacturer's instructions. Three different biological replicates were prepared. The integrity and concentration of RNA were determined using a Bioanalyzer system (RIN > 6) (Agilent Technologies) and a Qubit RNA broad-range assay kit (Life Technologies), respectively. To construct the sequencing library, 1.5 μg of total RNA from each sample was used as input for the TruSeq RNA sample prep kit ver. 2 (Illumina). The libraries were validated, quantified using the Bioanalyzer and the library qPCR quantification method, multiplexed with equal ratios, and loaded into the flow cell of the Illumina MiSeq Reagent Kit ver. 3 (2 × 75 runs). Sequencing was performed using a MiSeq Sequencer system (Illumina) and a total of 3 Gb (40 M paired-end reads) of sequencing data were generated (Q_30_ > 98%). The RNA-Seq data have been deposited to the Sequence Read Archive (SRA) under accession number SRP134977.

### Transcriptomic data analysis

All analyses were performed using the CLC Genomics Workbench ver. 8 module (Qiagen). After quality and adapter trimming, the raw reads were mapped to the rice reference genome using a gene model annotation file from the Michigan State University rice genome annotation project database ver. 7 (http://rice.plantbiology.msu.edu/). The gene expression levels were determined in units of fragments per kilobase of exon model per million mapped reads (FPKM) normalized values (Mortazavi et al., 2008). For statistical analysis, *t*-tests and Baggerley's tests were performed on the original and normalized read counts. In addition, several relevant values for analysis, such as *p*-values, corrected *p*-values for multiple correction, and test statistics, were calculated in the “multi-group comparison” option. Differentially expressed genes were those with a *p* < 0.05, corrected *p*-value of false discovery rate (FDR) < 0.05, and absolute fold-change value > 1.5 from pair-wise comparisons of FPKM values among different samples.

### Recombinant protein expression and gel retardation assay

Recombinant protein expression was conducted as described previously (Byun et al., 2015) with minor modifications. Full-length coding region of *DaCBF4* was inserted into the pProEx-HTa protein expression vector (Invitrogen, Carlsbad, CA, USA). Recombinant protein expressed in *E. coli* Rosetta (DE3) cells was purified by affinity chromatography using Ni-NTA agarose (Qiagen) according to the manufacturer's protocols. Purified protein was concentrated with Amicon Ultra-15 Centrifugal Filter Units (Merck). To investigate the characteristics of DaCBF4 as a transcription factor, gel retardation assays was conducted. The DNA fragments containing the CRT/DRE core repeat or low-temperature responsive element (LTRE) sequence were labeled with ^32^P-dCTP and incubated with recombinant DaCBF4 proteins in binding buffer [10 mM Tris HCl, pH 8.0, 50 mM NaCl, 1 mM EDTA, 1 mM DTT, and 5% glycerol (v/v)]. After incubation for 10 min on ice, components of the reaction mixtures were separated on 6% non-denatured polyacrylamide gels in 0.5× Tris-borate EDTA buffer. Gel was dried and visualized by autoradiography.

## Results

### Identification and characterization of DaCBF4

As the overexpression of barley *HvCBF4* and wheat *TaCBF14* (group IV) increased the cold-stress tolerance of transgenic rice and barley plants, respectively (Oh et al., 2007; Soltész et al., 2013), we cloned the *D. antarctica* cDNA sequence encoding the CRT-DREB-binding factor protein based on its sequence homology with *HvCBF4* (Lee et al., 2013b). The gene was designated *DaCBF4*, and the sequence was submitted to GenBank (KM978992).

*DaCBF4* encodes a protein of 216 amino acids with a conserved AP2 DNA-binding domain. To examine its phylogenetic relationships with CBF proteins from other monocot plants, we compared the amino acid sequence of DaCBF4 with those of 37 hexaploid wheat, 9 einkorn wheat, 17 barley, 9 rice, and 1 *D. antarctica* CBF homologs (Badawi et al., 2007; Byun et al., 2015). All members of the monocot CBF family were divided into five groups; DaCBF4 belonged, as expected, to group IV (Supplementary Figure 1). In the AP2 domain and flanking regions, DaCBF4 shares 70–75, 76–78, 67–79, 75–91, and 56–65% sequence identities with the members of groups I, II, III, IV, and V, respectively.

### DaCBF4 is a nuclear-localized transcription factor

**Figure 1:**
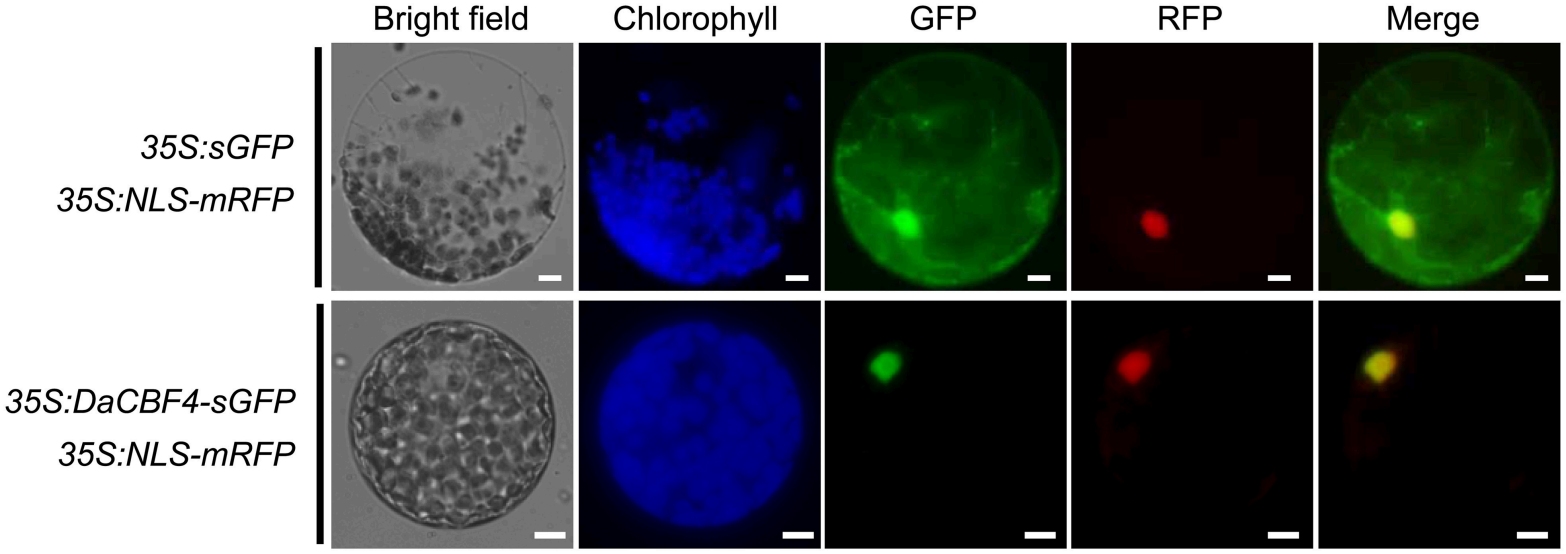
Subcellular localization of DaCBF4. Tobacco (*Nicotiana benthamiana*) leaves were co-infiltrated using *Agrobacterium* that contained the 3*5S:DaCBF4-sGFP* or *35S:sGFP* construct. NLS-mRFP was used as a nuclear marker protein. Scale bars = 5 μm.

Transcription factors must localize to the nucleus to induce the expression of downstream target genes. To examine the cellular localization of DaCBF4, an *in vivo* cell targeting experiment was performed. The *sGFP* gene was fused in-frame to the 3′ end of the DaCBF4-coding region under the control of the CaMV 35S promoter. *DaCBF4-sGFP* and *NLS-mRFP* (a nuclear marker) constructs were co-expressed in tobacco (*N. benthamiana*) leaves using the *Agrobacterium*-mediated infiltration method. The protoplasts were extracted and visualized by fluorescence microscopy. The fluorescence signal of sGFP was uniformly distributed throughout the cell (Figure 1). In contrast, the DaCBF4-sGFP fusion protein was found primarily in the nucleus, and its signal merged with that of NLS-mRFP, indicating that DaCBF4 is targeted to the nucleus of plant cells.

### Induction of *DaCBF4* expression in *D. antarctica* by cold and drought stresses

**Figure 2:**
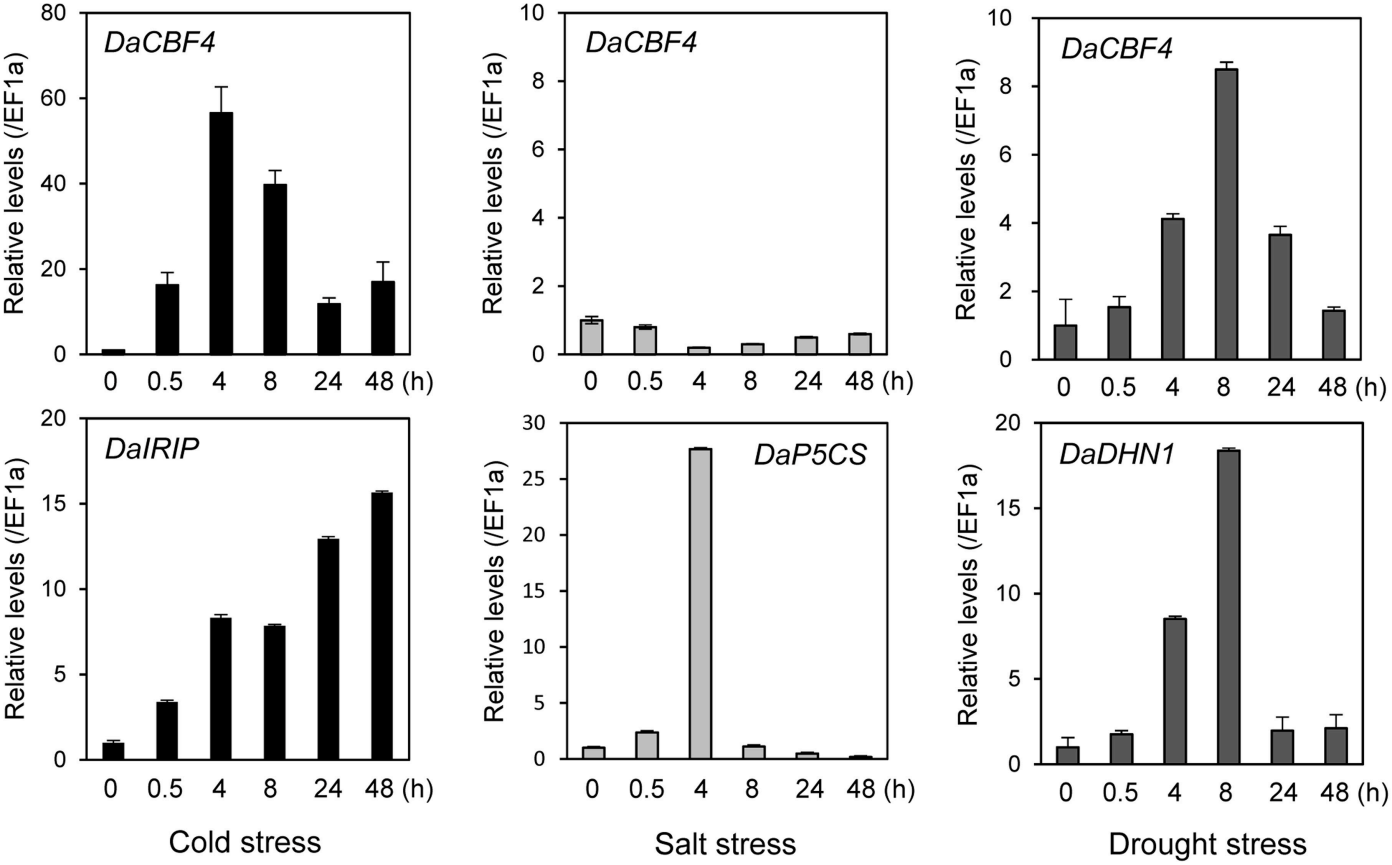
Expression of *DaCBF4* in *D. antarctica* under various abiotic stress conditions. The *DaCBF4* expression levels were measured by RT-qPCR with total RNA from *D. antarctica* leaves under cold (4°C), salt (150 mM NaCl), or drought (air dried on a filter paper at 15°C) stress. The *D. antarctica EF1a* gene was used as an internal control for normalization. The expression level of *DaCBF4* grown on normal MS at 15°C was used as a control (calibrator for quantification) and was assumed as 1. Error bars represents standard deviation of means (*n* = 3). The *DaIRIP, DaP5CS*, and *DaDHN1* genes were positive controls for cold, salt, and drought stresses, respectively. DNA sequences of gene-specific primers used for RT-qPCR are shown in Supplementary Table 2.

The expression of numerous monocot *CBF* genes is reportedly induced in response to abiotic stresses in plants (Dubouzet et al., 2003; Vágújfalvi et al., 2005; Byun et al., 2015). To investigate the effects of abiotic stresses on the *DaCBF4* transcript level in Antarctic hairgrass, *DaCBF4* mRNA levels were analyzed by RT-qPCR using total RNA isolated from leaves of *D. antarctica* plants subjected to cold (4°C), salt (150 mM NaCl), or drought (air-dried on filter paper) stress. The *DaCBF4* transcript level increased in response to cold-stress treatment. *DaCBF4* expression was induced from 30 min after cold stress, peaked at 4 h, and then declined but remained high until 48 h. Drought stress resulted in a similar pattern of *DaCBF4* expression. In contrast, salt stress did not activate the transcription of *DaCBF4* (Figure 2). The *DaIRIP, DaP5CS*, and *DaDHN1* genes were used as positive controls for the cold, salt, and drought stresses, respectively.

### Generation and molecular analysis of *Ubi:DaCBF4* transgenic rice plants

Genetic transformation is widely used to investigate the functional roles of specific genes in plants. However, transformation of genes into *D. antarctica* is not yet possible. Therefore, we overexpressed *DaCBF4* in rice, a monocot model plant of the same family as *D. antarctica*.

**Figure 3:**
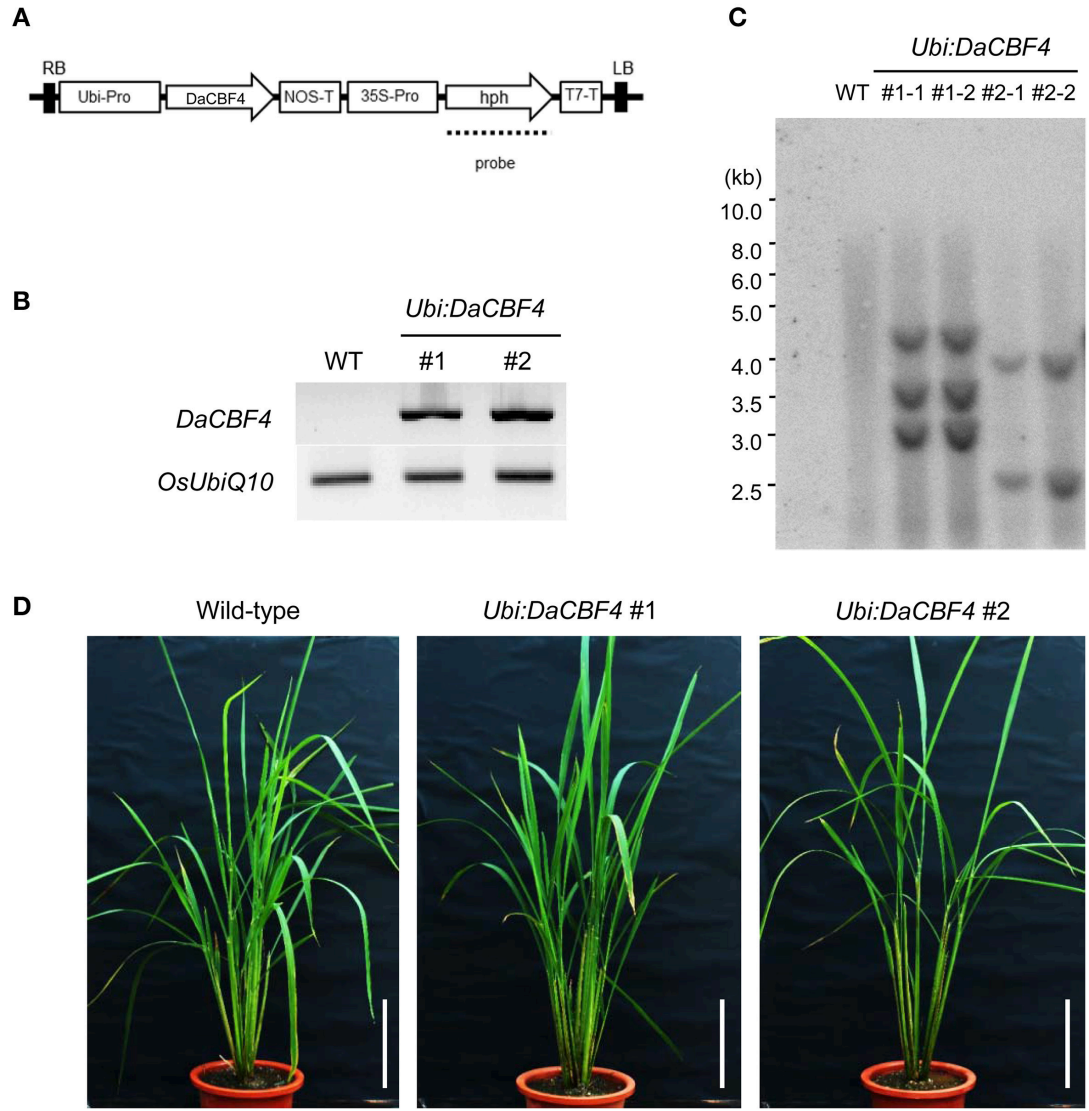
Generation and molecular characterization of *Ubi:DaCBF4* transgenic rice plants. **(A)** Schematic representation of the binary vector construct used for *DaCBF4* overexpression under the control of the maize ubiquitin promoter. RB, right border; Ubi-Pro, ubiquitin promoter; NOS-T, terminator sequence from nopaline synthase gene; 35S-Pro, CaMV 35S promoter; hph, hygromycin B phosphotransferase; T7-T, T7 terminator; LB, left border. **(B)** Semi-quantitative RT-PCR analysis of 6-week-old wild-type and two independent *Ubi:DaCBF4* T2 transgenic plants (lines #1 and #2). **(C)** Genomic Southern blot analysis. Total leaf genomic DNA was isolated from wild-type and T2 *Ubi:DaCBF4* transgenic rice plants. DNA was digested by *Bam*HI and hybridized with ^32^P-labeled hph probe under high-stringency conditions. **(D)** Overall morphology of 2-month-old wild type and T2 *Ubi:DaCBF4* transgenic (independent lines #1 and #2) rice plants. Rice plants were grown under greenhouse conditions. Scale bars = 10 cm.

We generated transgenic rice plants constitutively overexpressing *DaCBF4* under the control of the maize ubiquitin (Ubi) promoter (Figure 3A). Semi-quantitative RT-PCR confirmed that two different *Ubi:DaCBF4* transgenic plants overexpressed *DaCBF4* (Figure 3B). DNA gel-blot analysis indicated that these *Ubi:DaCBF4* transgenic plants were independent lines (Figure 3C). Transgenic *Arabidopsis* plants overexpressing *AtCBF1, AtCBF2*, or *AtCBF3* at high levels exhibit the dwarf phenotype (small stature and slow growth) under normal conditions (Gilmour et al., 2004). Moreover, in cereals, transgenic rice overexpressing *OsDREB1* and transgenic barley overexpressing *TaCBF14, TaCBF15*, or *HvCBF2A*, showed moderate retarded development under normal condition compared to wild-type plants (Ito et al., 2006; Soltész et al., 2013; Jeknić et al., 2014). In contrast, the two independent *Ubi:DaCBF4* transgenic lines (#1 and #2) exhibited wild-type vegetative growth under our experimental conditions (Figure 3D).

### Effect of *DaCBF4* overexpression on abiotic stress tolerance in transgenic rice plants

**Figure 4:**
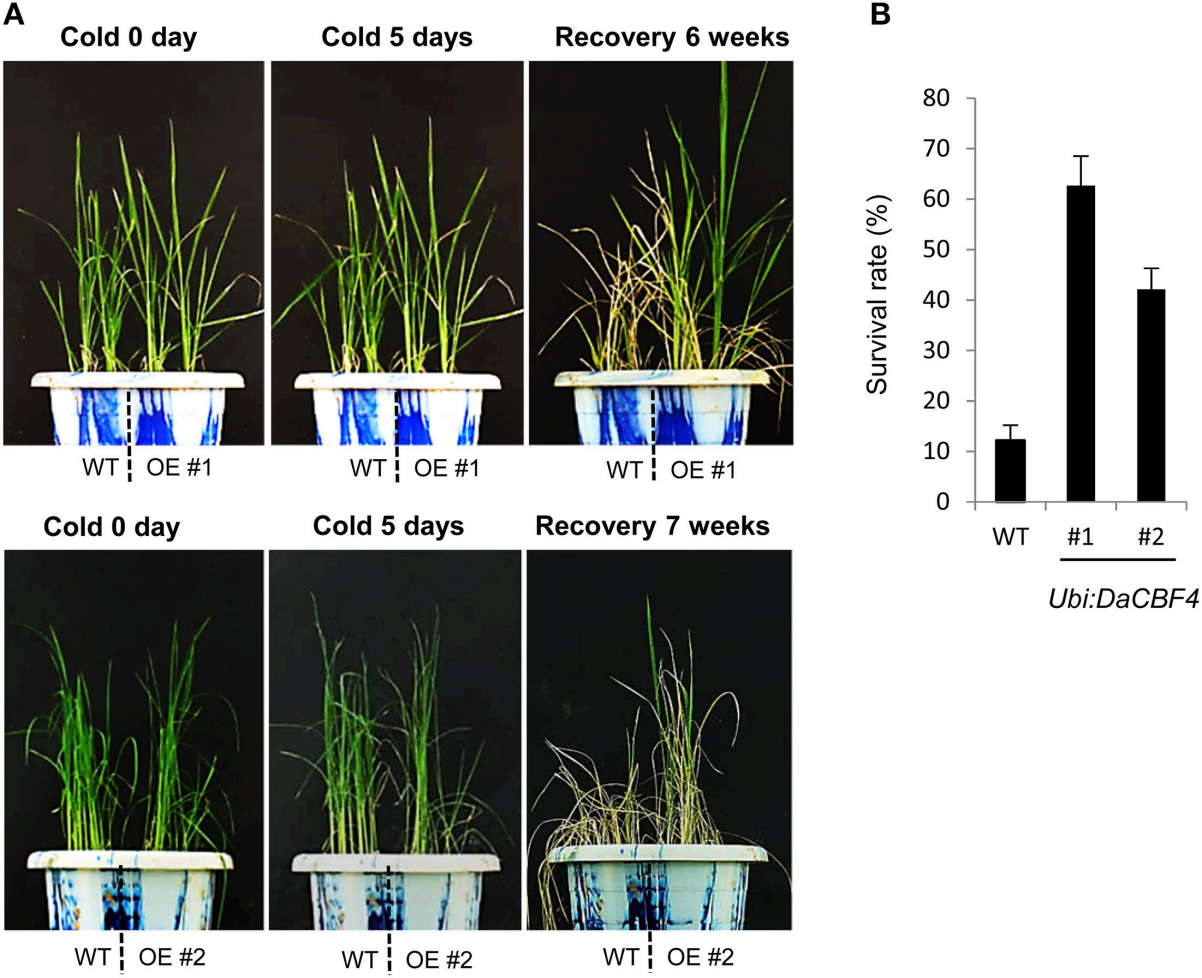
Comparison of cold-stress tolerance between wild-type (WT) and *Ubi:DaCBF4* transgenic plants. **(A)** Six-week-old wild type (WT) and T2 *Ubi:DaCBF4* (line #1 and #2) rice plants were subjected to cold treatment at 4°C for 5 days, recovered to normal condition at 28°C, and then compared their survival rates.**(B)** Survival rates of WT and two independent transgenic lines under cold-stress conditions. Results are expressed as means from four independent experiments and 11 plants were used for each experiment. Error bars represents standard deviation of means of independent experiments.

Overexpression of heterologous *CBF* genes in rice results in tolerance to cold (Byun et al., 2015), drought and high salt (Oh et al., 2005; Cui et al., 2011), or cold, drought, and high salt (Oh et al., 2007; Xu et al., 2011). To investigate whether overexpression of *DaCBF4* is correlated with tolerance to abiotic stresses in transgenic plants, we assessed the effect of cold stress on *Ubi:DaCBF4* transgenic rice plants. Wild-type and *Ubi:DaCBF4* transgenic plants were grown at 28°C for 6 weeks in the growth room and transferred to the cold room (4°C). After 5 days of cold-stress conditions, wild-type plants showed wilting and did not recover after removal from the cold-stress conditions, while most of the *Ubi:DaCBF4* transgenic rice plants recovered, appeared healthy, and continued to grow (Figure 4A). The survival rate of the wild-type rice plants was 11.8% (12 of 101), and the survival rates of *Ubi:DaCBF4* lines #1 and #2 were 63.6% (35 of 55) and 41.3% (19 of 46), respectively (Figure 4B).

As *DaCBF4* expression was also induced by drought (Figure 2), we assessed the tolerance of wild-type and *Ubi:DaCBF4* rice plants to drought stress. Six-week-old wild-type and *Ubi:DaCBF4* plants were grown without a water supply for 9 days. Both wild-type and *DaCBF4*-overexpressing plants were severely wilted, and most plants did not recovered and died even after re-watering. Next, we compared the salt tolerance of the wild-type and *Ubi:DaCBF4* transgenic lines. Six-week-old plants were irrigated with water supplemented with 200 mM NaCl for 10 days, re-watered with tap water, and their growth patterns were observed. Under this condition, both wild-type and transgenic plants were almost entirely bleached and unable to recover. Therefore, constitutive expression of *DaCBF4* increased the tolerance of rice to cold stress, but had no effect on the tolerance to dehydration or salt stress.

### Transcriptome analysis of *Ubi:DaCBF4* transgenic plants under cold-stress conditions

As *DaCBF4*-overexpressing rice plants were more tolerant to cold stress than wild-type plants, we next performed a transcriptomic analysis of *Ubi:DaCBF4* and wild-type plants to identify the downstream target genes of DaCBF4 responsible for the enhanced cold tolerance. We conducted RNA-seq analysis using the total RNA from 6-week-old leaves of wild-type and *Ubi:DaCBF4* (transgenic line #1) plants grown under normal (before cold treatment) and cold-stress (1 and 6 days at 4°C) conditions. Transgenic line #1 was selected for RNA-seq analysis because it displayed a higher survival rate after cold stress than line #2 (Figure 4). The raw reads were mapped to the rice reference genome. The expression values were measured in FPKM and the differentially expressed genes (DEGs) were determined.

We reported previously that constitutive expression of *DaCBF7*, one of the *CBF* genes of *D. antarctica*, enhanced the cold-stress tolerance of transgenic rice plants by modulating the expression levels of downstream genes (Byun et al., 2015). Because the *DaCBF4*-overexpressing plants showed a cold-specific phenotype similar to that of the *DaCBF7*-overexpressing plants, we identified the genes responsible for the increased the cold tolerance by screening for genes up- or down-regulated in both transgenic rice plants.

### Genes upregulated under normal conditions

**Figure 5:**
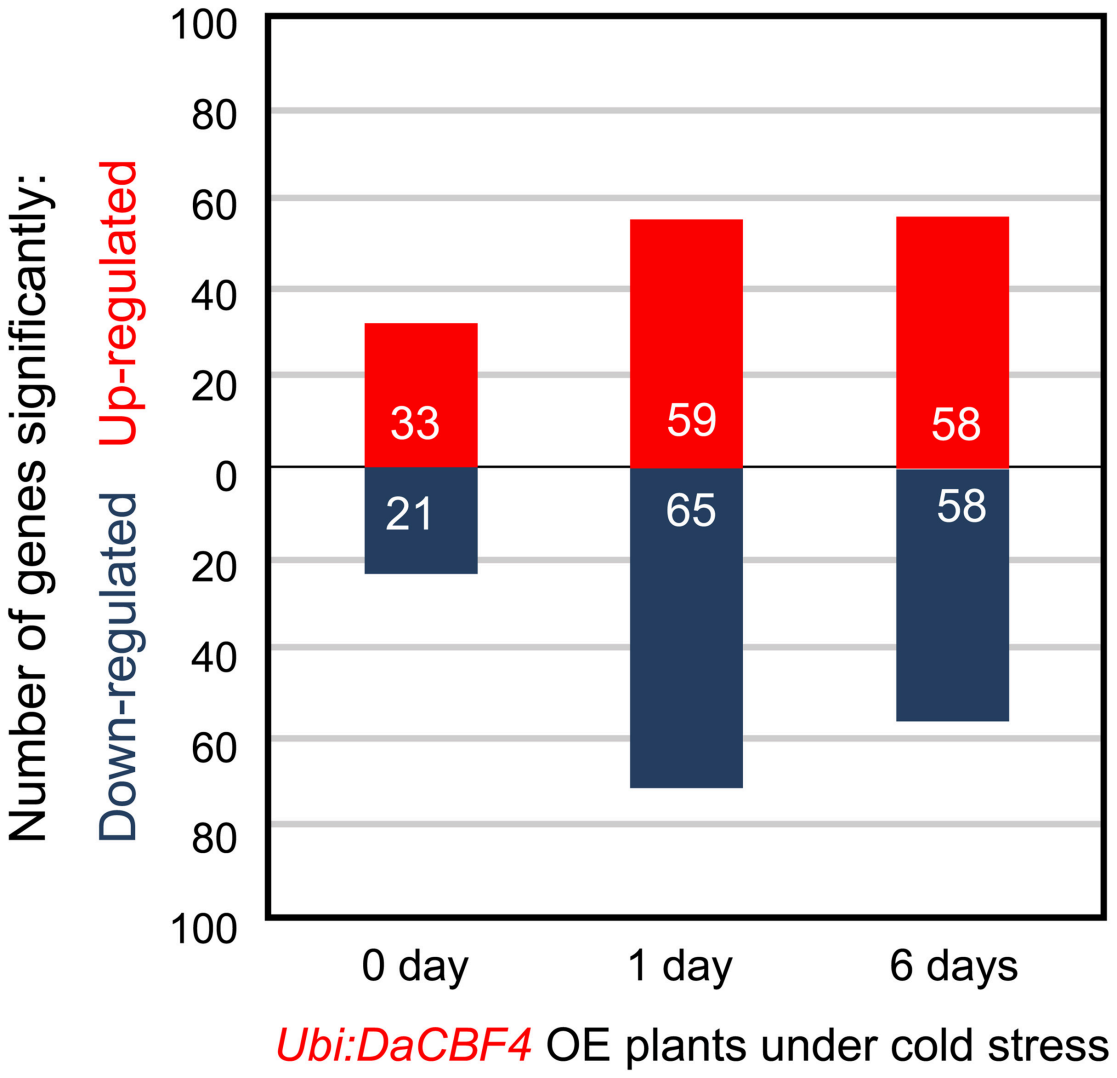
Transcriptome analysis with RNA-seq of the cold-stress response of *Ubi:DaCBF4* transgenic plants. Number of differentially expressed genes in *Ubi:DaCBF4* line #1 relative to the expression level of wild-type (WT) at each time point (0, 1, and 6 days) of cold treatment (*p* < 0.05, corrected *p*-value of FDR < 0.05).

Under normal growth conditions, 33 genes were upregulated in *Ubi:DaCBF4* line #1 compared to wild-type rice plants (Figure 5 and Supplementary Table 3). This indicated that constitutive expression of *DaCBF4* influenced the gene expression profile of transgenic plants even before cold treatment. Among them, nine genes were also upregulated in *Ubi:DaCBF7* plants compared to wild-type plants (Table 1), including those encoding a protease inhibitor family protein (*BBTI12*, Os01g04050) and non-specific lipid transfer proteins (*LTPL114*, Os03g01300; *LTPL12*, Os12g02320). The expression of three genes annotated as expressed proteins was highly induced in both transgenic plants. Furthermore, the induction of the expression of the genes encoding dirigent (Os11g10850), thaumatin (Os12g43380), and ribonuclease T2 family domain-containing protein (Os09g36680) differed between the two transgenic plants under normal conditions.

**Table 1:** List of differentially induced genes in both transgenic rice plants *Ubi:DaCBF4* and *Ubi:DaCBF7* under normal control or cold stress conditions when compared to wild type plants.

**Locus ID**	**Annotations—Description**	**RNA-seq analysis; Baggerley's test**			
		***Ubi:DaCBF*4 vs. wild-type**		***Ubi:DaCBF7* vs. wild-type**	
		**Fold change**	**FDR *p*-value correction**	**Fold change**	**FDR *p*-value correction**
**NORMAL CONDITION**					
Os11g10850	Dirigent	15.42	3.26E−02	210	1.09E-03
Os12g43380	Thaumatin	5.17	1.54E−04	11.21	0
Os09g36680	Ribonuclease T2 family domain containing protein	3.06	0	2.89	0
Os01g04050	BBTI12—Bowman-Birk type bran trypsin inhibitor	2.26	2.43E−04	3.90	1.50E−03
Os12g02320	LTPL12—Protease inhibitor family protein	1.60	1.51E−02	1.94	1.11E−10
Os03g01300	LTPL114—Protease inhibitor family protein	1.60	2.89E−11	11.98	2.92E−08
Os10g22630	Expressed protein	614.27	1.13E−11	453.35	0
Os03g02470	Expressed protein	211.86	1.41E−02	457	8.73E−09
Os01g15270	Expressed protein	1.77	9.15E−08	2.04	4.90E−06
**COLD STRESS, 1 DAY**					
Os02g43970	AP2 domain containing protein	30.60	0	385.13	2.84E−07
Os03g45280	Dehydrin (OsLEA24)	5.22	0	1.36	1.60E−11
Os09g35030	Dehydration-responsive element-binding protein	2.91	1.81E−05	1.77	9.62E−11
Os08g37670	Plastocyanin-like domain containing protein	2.82	0	4.26	0
Os01g09220	Transposon protein putative CACTA En/Spm sub-class	2.16	6.39E−07	3.44	0
Os07g48630	Ethylene-insensitive 3	2.06	7.36E−12	1.39	2.01E−02
Os01g67480	Helix-loop-helix DNA-binding domain containing protein	2.00	1.97E−03	1.67	2.92E−02
Os09g36680	Ribonuclease T2 family domain containing protein	1.69	3.44E−04	3.56	0
Os01g21250	Late embryogenesis abundant protein (OsLEA9)	1.59	0	1.53	0
Os04g58710	AMP-binding domain containing protein	1.22	2.21E−07	1.97	0
Os01g06882	Expressed protein	1154.57	3.62E−03	378	3.61E−07
Os04g01330	Expressed protein	535.30	1.17E−06	385.13	0
Os10g22630	Expressed protein	279.51	9.38E−05	388.62	0
Os03g02470	Expressed protein	191.20	6.65E−03	291	2.63E−05
Os06g09900	Expressed protein	1.79	1.32E−10	1.25	9.13E−03
**COLD STRESS, 6 DAY**					
Os02g43970	AP2 domain containing protein	106.23	2.94E−08	99.36	1.90E−05
Os02g03280	Transmembrane BAX inhibitor motif-containing protein	3.84	0	1.27	1.26E−03
Os10g33800	Lactate/malate dehydrogenase	2.26	6.68E−04	1.82	8.47E−10
Os03g45280	Dehydrin (OsLEA24)	1.93	6.41E−11	1.72	7.26E−11
Os04g16770	Photosynthetic reaction center protein	1.81	0	1.39	0
Os09g25320	Ubiquitin family protein	1.79	4.38E−02	1.45	2.82E−03
Os08g35420	Photosynthetic reaction center protein	1.50	0	2.67	0
Os01g68300	Expressed protein	2.99	1.30E−02	2.90	0
Os12g39930	Expressed protein	1.62	0	5.96	0
Os10g21190	Expressed protein	1.59	0	4.66	0

*Results for Ubi:DaCBF7 were adopted from Byun et al. (2015)*.

### Genes upregulated under cold-stress conditions

After 1 day of cold-stress conditions, 59 genes were upregulated in the *Ubi:DaCBF4* transgenic rice plants compared to the wild type (Figure 5 and Supplementary Table 4). Of these, 15 were upregulated in both *Ubi:DaCBF4* and *Ubi:DaCBF7* after 1 day of cold-stress conditions (Table 1). The gene encoding AP domain-containing protein (Os02g43970) exhibited the greatest fold-change in expression in both transgenic lines compared to wild-type plants, except for five genes annotated as expressed proteins. This indicates that activation of the expression of downstream *COR* genes in transgenic plants is a multi-step process. Three transcription factor-encoding genes, dehydration-responsive element-binding protein (Os09g35030), ethylene-insensitive 3 (Os07g48630), and helix-loop-helix DNA-binding domain containing protein (Os01g67480), were upregulated, suggesting a role in the cold tolerance of rice. In addition, the expression of two late embryogenesis-abundant protein (LEA) genes encoding OsLEA9 (Os01g21250) and OsLEA24 (Os03g45280) were induced under cold-stress conditions. The expression of *OsLEA24* was induced after 1 and 6 days of cold-stress conditions, but *OsLEA9* expression was induced only after 6 days. The expression levels of *OsBI* (transmembrane BAX inhibitor motif-containing protein; Os02g03280) and *OsMDH* (lactate/malate dehydrogenase; Os10g33800) were increased after 6 days of cold-stress conditions.

### Genes downregulated in transgenic rice plants

In total, 130 genes were downregulated in *Ubi:DaCBF4* under normal and cold-stress conditions (Supplementary Table 5). To identify enriched Gene Ontology (GO) terms for these genes, over-represented GO categories were analyzed using AgriGO (Fisher's exact test *p* < 0.05) (Tian et al., 2017).

In the Biological Process category, the GO terms photosynthesis, generation of precursor metabolites and energy, and translation were significantly enriched in downregulated genes in *Ubi:DaCBF4* plants; the most highly enriched GO term was photosynthesis with the lowest corrected *p*-value of FDR, 2.21e−15 (Supplementary Figure 2). Six of the 14 genes included in the GO term photosynthesis were significantly downregulated in *Ubi:DaCBF7* plants under cold-stress compared to normal conditions. These comprised the genes encoding chlorophyll A-B binding protein (Os01g41710 and Os07g37240), photosynthetic reaction center protein (Os08g35420), photosystem II 10 kDa polypeptide chloroplast precursor (Os08g10020), photosystem II 44 kDa reaction center protein (Os04g16874), and photosystem II D2 protein (Os04g16872) (Table 2).

**Table 2:** List of differentially down-regulated genes belonging to GO term “Photosynthesis” (GO:0015979) in both transgenic rice plants *Ubi:DaCBF4* and *Ubi:DaCBF7*under normal control or cold stress condition when compared to wild type plants.

**Locus ID**	**Annotations—description**	**RNA-seq analysis; Baggerley's test**			
		***Ubi:DaCBF4* vs. wild-type**		***Ubi:DaCBF7* vs. wild-type**	
		**Fold change**	**FDR *p*-value correction**	**Fold change**	**FDR *p*-value correction**
**NORMAL CONDITION**					
Os01g41710	Chlorophyll A-B binding protein	−1.760	1.782E−22	−2.044	0
Os08g10020	Photosystem II 10 kDa polypeptide chloroplast precursor	−1.391	8.340E−06	−1.591	2.654E−120
**COLD STRESS, 1 DAY**					
Os08g35420	Photosynthetic reaction center protein	−2.455	2.951E−72	−10.319	3.584E−189
Os07g37240	Chlorophyll A-B binding protein	−2.367	2.021E−06	−1.244	3.942E−10
Os04g16874	Photosystem II 44 kDa reaction center protein	−1.741	9.320E−06	−5.394	1.856E−16
Os04g16872	Photosystem II D2 protein	−1.428	1.597E−02	−4.161	6.194E−19

*Results for Ubi:DaCBF7 were adopted from Byun et al. (2015)*.

### Validation of DEGs in *Ubi:DaCBF4* transgenic plants

**Figure 6:**
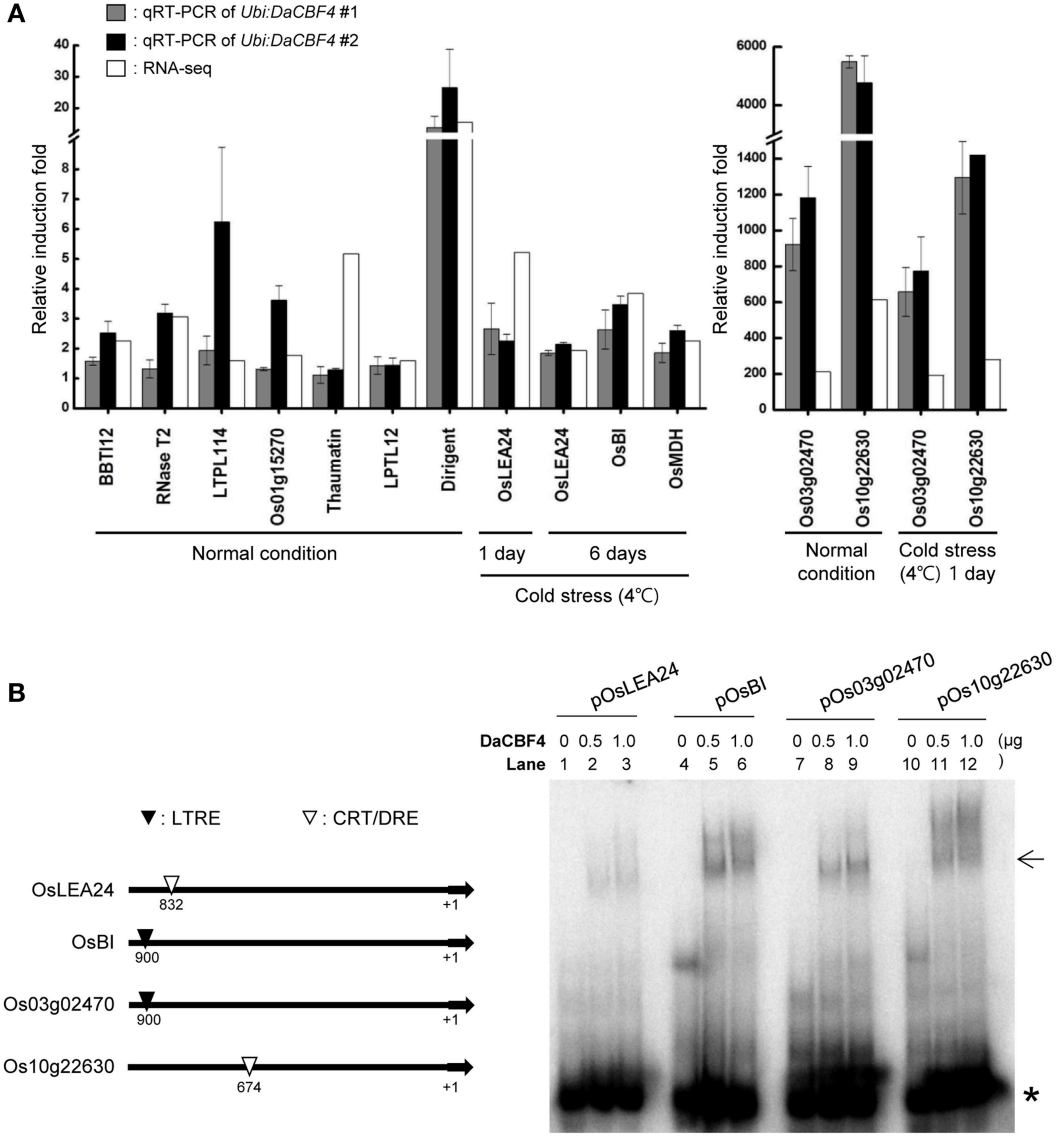
Validation of the gene expression profiles obtained in RNA-seq by RT-qPCR and gel retardation analysis. **(A)** Light-grown, 6-week-old wild-type and *Ubi:DaCBF4* plants were subjected to cold (4°C) stress for 1 day and 6 days, respectively. DEGs commonly up-regulated in *Ubi:DaCBF4* and *Ubi:DaCBF7* transgenic plants under normal condition or cold stress condition were confirmed by RT-qPCR using the gene-specific primers listed in Supplementary Table 2. Data represent the fold induction of each gene in *Ubi:DaCBF4* #1 and #2 plants relative to wild type plants. Gray bars show relative gene expression of *Ubi:DaCBF4* #1 normalized to the level of *OsActin* mRNA as an internal control, which indicate means ± *SD* from three biological replicates. In addition, black bars indicate relative induction fold of *Ubi:DaCBF4* #2, which indicate means ± *SD* from biological duplicates. White bars represent fold change of the selected genes in RNA-seq. **(B)** Gel retardation assay. (Left panel) Schematic presentation of four DaCBF4-induced target genes. +1 indicates a transcription initiation site. Putative CRT/DRE and LTRE motifs are indicated by ∇ and ▼, respectively. (Right panel) Gel retardation assay was performed with the ^32^P-radiolabeled promoter sequence of *OsLEA24* (lanes 1-3), *OsBI* (lanes 4-6), *Os03g02470* (lanes 7-9), *Os10g22630* (lanes 10-12). Each set of lanes contained 0, 0.5, or 1.0 μg of bacterially-expressed, full-length His-DaCBF4 recombinant protein, respectively. Arrow and asterisk (*) indicate positions of DNA-DaCBF4 complex and free probe, respectively.

To validate the DEG results, 12 genes that were differentially expressed in *Ubi:DaCBF4* transgenic plants under normal and cold-stress conditions were subjected to RT-qPCR (Figure 6A). Under normal conditions, the expression patterns of nine genes—including *BBTI12* (Os01g04050), *RNase T2* (Os09g36680), *LTPL114* (Os03g01300), *Thaumatin* (Os12g43380), *LTPL12* (Os12g02320), *Dirigent* (Os11g10850)—and three unknown genes—Os01g15270, Os03g02470, and Os10g22630—were in overall agreement with those determined by RNA-seq. The fold-changes in expression determined by RNA-seq and RT-qPCR differed slightly for *LTPL114, Thaumatin*, and Os10g22630, but their expression was increased in *Ubi:DaCBF4* line #1 and line #2 compared to wild-type plants. Under cold-stress conditions, the expression patterns of five genes—including *OsLEA24* (Os03g45280), *OsBI* (Os02g03280), *OsMDH* (Os10g33800)—and two unknown genes—Os03g02470 and Os10g22630—were in agreement with those determined by RNA-seq, and the expression of these genes was higher in independent two lines of *Ubi:DaCBF4* compared to wild-type plants.

Promoter analysis represented that *OsLEA24* and *Os10g22630* contain putative CRT/DRE cis-acting elements in their promoter regions (Figure 6B). In addition, the upstream regions of the *OsBI* and *Os03g02470* have a putative low temperature responsive element (LTRE). To investigate the function of DaCBF4 as a transcription factor, we conducted gel retardation analysis with ^32^P-labeled promoter regions of the target gene promoter. As shown in Figure 6B, the His-DaCBF4 protein bound to DNA and formed nucleoprotein complex. These results are in agreement with the fact that the above-mentioned genes are regulated by ectopic expression of *DaCBF4* in rice plants.

### Differential expression of AP2 genes in *Ubi:DaCBF4* and *Ubi:DaCBF7*

In addition to searching for genes responsible for increased cold tolerance, investigating the specificity of DaCBF4 to its downstream targets is important for understanding the molecular mechanism of regulation for downstream genes by the group IV CBF. We could make a list of 72 genes which were upregulated exclusively in *Ubi:DaCBF4* under cold stress condition, compared to *Ubi:DaCBF7*. When over-represented GO categories were analyzed using AgriGO (Fisher's exact test *p* < 0.05) (Tian et al., 2017), the GO term regulation of metabolic process was the only significantly enriched one (corrected *p*-value of FDR = 0.022). Notably, seven genes included in this group were encoding AP2 domain containing protein (Supplementary Table 6). For comparative analysis, we also identified 7 more AP2 genes, which were induced exclusively in *Ubi:DaCBF7* under cold stress. As a result of promoter analysis, we could identify 9 DRE/CRT and 4 LTRE elements from DaCBF4 induced 7 AP2 genes, and 5 DRE/CRT and 8 LTRE elements from DaCBF7 induced 7 AP2 genes, which imply that different CBF homolog protein may prefer different type of *cis*-element in promoters of target genes.

## Discussion

### Cloning and characterization of *DaCBF4* from *D. antarctica*

We isolated *DaCBF4* from the Antarctic plant *D. antarctica* (Poaceae; Pooideae). Phylogenetic analysis revealed that DaCBF4 is grouped within the cereal CBF group IV (Badawi et al., 2007). Because cereal CBF group IV is found only in the Pooideae, members of this group are considered to have evolved during the recent translocation of these plants from tropical to temperate habitats, suggesting possible roles in adaptation to low temperature (Badawi et al., 2007). RT-qPCR analyses revealed that the expression levels of *DaCBF4* in *D. antarctica* under cold and drought stresses were increased, and cold stress resulted in an increase in expression of greater magnitude than did drought stress (Figure 2). Induction of CBF IV group gene expression by cold stress also occurs in barley, wheat, and *Brachypodium distachyon* (Kume et al., 2005; Skinner et al., 2005; Chen et al., 2016). Together with the cellular localization of *35S:DaCBF4-sGFP* in the nucleus of tobacco protoplasts (Figure 1), DNA binding affinity of DaCBF4 (Figure 6B) represented possible roles of this protein as a transcription factor in Antarctic hairgrass. The considerable induction of *DaCBF4* expression under cold stress suggests that *DaCBF4* regulates development of cold tolerance in *D. antarctica* during adaptation to the low-temperature environment of the Antarctic.

### Phenotype of *Ubi:DaCBF4* plants under normal and cold-stress conditions

We generated *CBF*-overexpressing transgenic plants to assess the roles of these genes in tolerance to abiotic stresses. Most of the transgenic plant lines exhibited enhanced tolerance to cold, drought, and salt stresses; this was mediated by modulating the expression of downstream stress-responsive genes (Lata and Prasad, 2011). Constitutive expression of *CBF* transcription factors generally enhances stress tolerance, but in some cases results in retarded normal growth (dwarf phenotype) and delayed flowering (Ito et al., 2006; Wei et al., 2017). In contrast, other *CBF*-overexpressing transgenic plants displayed no differences in phenotype under normal growth conditions (Oh et al., 2007; Xu et al., 2011; Byun et al., 2015). In this study, constitutive expression of *DaCBF4* from *D. antarctica* enhanced cold-stress tolerance without affecting development. The distinct effects of *CBF* overexpression on vegetative growth may be due to different generation of transgenic plants analyzed (Oh et al., 2007). However, the molecular mechanisms underlying the different growth phenotypes of the various transgenic plant lines remain to be elucidated.

### Comparative RNA-Seq analysis of *Ubi:DaCBF4* and *Ubi:DaCBF7*

Overexpression of *CBF* reportedly enhances the cold tolerance of barley, rice, and wheat (Ito et al., 2006; Oh et al., 2007; Gao et al., 2009; Morran et al., 2011; Xu et al., 2011). However, only a few studies have performed high-throughput transcriptome analysis of downstream genes regulated by overexpressed CBF transcription factors, all of them in rice (Oh et al., 2005, 2007; Ito et al., 2006). We reported previously that the overexpression of *DaCBF7*, a *CBF* homolog from *D. antarctica*, resulted in enhanced tolerance to cold in rice, similar to *DaCBF4* in this study. Moreover, the transcriptome of *Ubi:DaCBF7* was analyzed by RNA-seq to identify downstream genes involved in promoting cold tolerance in transgenic rice plants (Byun et al., 2015). Therefore, in this study we focused on downstream target genes regulated by both *DaCBF7* and *DaCBF4* that enhance the cold tolerance of rice.

### Genes upregulated in *Ubi:DaCBF4* and *Ubi:DaCBF7* plants under normal conditions

Nine genes were upregulated in both *Ubi:DaCBF4* and *Ubi:DaCBF7* transgenic rice plants under normal conditions (Table 1), the majority are abiotic stress-responsive genes in various plant species. *LTPL12* (Os12g02320) and *LTPL114* (Os03g01300) encode non-specific lipid transfer proteins (LTPs) involved in lipid metabolism. In addition, *LTP* expression is induced in plant cells upon exposure to abiotic stresses. The bromegrass LTP gene *BG-14* was strongly induced during cold acclimation (Wu et al., 2004), and the expression of two barley *LTP4* genes, *HvLtp4.2*, and *HvLtp4.3*, was increased under cold-stress conditions (Molina et al., 1996). Overexpression of the pearl millet *DREB2A* transcription factor gene in tobacco and of the rice *DREB1A* gene in rice plants enhanced abiotic stress tolerance and induced the expression of *LTP* homologs under cold-stress conditions (Ito et al., 2006; Agarwal et al., 2010). The LTP-mediated resistance of plants to cold is associated with a decrease in thylakoid membrane lipid fluidity (Sror et al., 2003). Dirigent (Os11g10850) encodes a protein that modulates lignin biosynthesis and cell wall metabolism during exposure to abiotic stresses (Paniagua et al., 2017). Dirigent proteins are implicated in modulation of lignification levels upon exposure to abiotic stresses. The expression of several of the DIR-like genes from *Brassica rapa* and the resurrection plant *Boea hygrometrica* (*BhDIR1*) was influenced by water and cold stress (Wu et al., 2009; Arasan et al., 2013). Therefore, modulation of plant cell wall metabolism and fluidity is crucial for the cold-stress tolerance of plants. However, the molecular mechanisms underlying the effects of dirigent proteins on plant abiotic stress tolerance are unclear.

BBTI12 (Os01g04050) belongs to the Bowman-Birk family, which consists of compound inhibitors comprising one to six inhibitor units that target serine proteases (Rawlings, 2010). *BBTI1* and *BBTI2*, rice BBTI homologs, are induced under normal conditions in barley *CBF4*- or *Arabidopsis CBF3*-overexpressing transgenic rice plants (Oh et al., 2005, 2007). Proteins are influenced by abiotic stressors and, are the major players in plant responses to stress (Kidrič et al., 2014); thus, BBTI-mediated fine control of protein degradation may be required for survival of plants exposed to abiotic stresses. Expression of *OsRNS4*, which encodes the ribonuclease T2 family domain-containing protein (Os09g36680) was increased in response to biotic stresses, such as insects and *Xanthomonas oryzae*, and abiotic stresses, such as wounding and high-salt conditions (Hillwig, 2009). Overexpression of *OsRNS4* enhanced tolerance to a high salt concentration, but the effect on cold tolerance was not evaluated (Zheng et al., 2014). Because OsRNS4 is an inactive RNase (Hillwig, 2009), its role in the response to abiotic stresses may be independent of its enzymatic activity. In this study, the expression of *Thaumatin*, a member of the pathogenesis-related protein 5 (PR5) family, was induced in transgenic rice plants. Moreover, the expression in winter wheat (*T. aestivum* L.) of *TaTLP*, which encodes a thaumatin-like protein (TLP), was increased during cold acclimation of wheat seedlings (Kuwabara et al., 2002). Overexpression of *ObTLP1*, a thaumatin-like protein from basil, in *Arabidopsis* enhances tolerance of plants to multiple abiotic stresses as well as the phytopathogenic fungi, *Scleretonia sclerotiorum* and *Botrytis cinerea* (Misra et al., 2016).

To summarize, DaCBF4 and DaCBF7 enhance cold-stress tolerance by activating the expression of target genes with roles in biotic and abiotic stress responses. Interestingly, most of the genes upregulated in both plants under normal conditions are reportedly responsive to both biotic and abiotic stresses. Therefore, the pathogen resistance of *Ubi:DaCBF4* and *Ubi:DaCBF7* plants warrants further investigation.

### Genes upregulated in *Ubi:DaCBF4* and *Ubi:DaCBF7* under cold-stress conditions

Fifteen and ten genes were upregulated in both transgenic plants in response to 1 day and 6 days of cold stress, respectively (Table 1). Among them, two were *LEA* (late embryogenesis abundant) genes, which respond to cold, drought, salinity, and ABA stresses during the vegetative stage of growth (Liu et al., 2017). *LEA* genes are abundant in plant genomes; the *Arabidopsis* and rice genomes harbor at least 51 and 34 *LEA* genes, respectively (Wang et al., 2007; Hundertmark and Hincha, 2008). LEA proteins are classified into seven groups according to their motifs (Battaglia et al., 2008). The diverse gene expression levels and protein subcellular distributions suggest that different LEAs are involved in the responses to diverse environmental stimuli (Hundertmark and Hincha, 2008; Candat et al., 2014). LEA proteins may be involved in protection of proteins or endomembrane structures under stress conditions (Koag et al., 2003; Drira et al., 2013). *LEA* expression enhances stress tolerance in transgenic plants (Kosová et al., 2014). Overexpression of wheat *WCI16* or *Arabidopsis Cor15A* leads to increased freezing tolerance in transgenic plants (Artus et al., 1996; Sasaki et al., 2014). Heterologous expression of individual *PmLEAs* genes in tobacco conferred enhanced tolerance to cold and drought stress (Bao et al., 2017). *OsDhn1*-overexpressing plants displayed enhanced drought- and salt-stress tolerance due to increased scavenging of reactive oxygen species (Kumar et al., 2014). Transcriptome analysis of *Ubi:DaCBF4* and *Ubi:DaCBF7* plants showed specific changes in two LEA proteins, OsLEA9 (group III) and OsLEA24 (group II [dehydrin], characterized by a K-segment). These genes have a DRE/CRT sequence in their upstream regions and may be directly regulated by DaCBF proteins (Byun et al., 2015). Elevated expression levels of *OsLEA9* and *OsLEA24* are positively correlated with cold-stress tolerance in transgenic rice plants. Overall, these results suggest that the *OsLEA9* and *OsLEA24* genes, which are downstream of *DaCBF*, can be used as target genes for genetic engineering of crops with enhanced stress tolerance.

In addition to cold responsive genes, the induction of a rice gene *Os02g43970*, encoding AP2 domain containing protein, was highly strong under cold condition, but was not significant in the normal condition (Table 1). Moreover, 7 more genes encoding AP2 domain containing protein were significantly upregulated exclusively in *Ubi:DaCBF4* under cold condition, but not in *Ubi:DaCBF7* under normal or cold conditions (Supplementary Table 6). Based on results that those *CBF* genes still can exhibit a cold responsive expression pattern, we can assume that their induction in *Ubi:DaCBF4* is caused by additive effect of *DaCBF4* overexpression and native *CBF* response to cold treatment. Similar phenomena were also observed in previous studies on transgenic barley overexpressing *TaCBF2*/*3, TaCBF14*/*15*, and *HvCBF2A* (Morran et al., 2011; Soltész et al., 2013; Jeknić et al., 2014).

For more investigation on difference of target specificities between two transgenes *DaCBF4* and *DaCBF7*, we examined *cis*-element sequences of two AP2 gene groups, DaCBF4 induced and DaCBF7 induced group. DaCBF4 induced AP2 genes had more DRE/CRT (9) and less LTRE elements (4) than DaCBF7 induced 7 AP2 genes with 5 DRE/CRT and 8 LTRE elements. Obviously further researches on the genome wide analysis of rice AP2 genes and their distribution profile of promoter elements are still necessary, activation of different subset of AP2 genes by DaCBF4 and DaCBF7 can be an indirect evidence of distinctive specificity and biological activity for each CBF protein in plants under abiotic stresses.

### Genes downregulated in *Ubi:DaCBF4* and *Ubi:DaCBF7* plants

In total, 130 genes were downregulated in the *Ubi:DaCBF4* transgenic line under normal and cold-stress conditions. Photosynthesis was the most enriched GO term (Supplementary Figure 2). Six genes encoding photosystem proteins were significantly downregulated in both *Ubi:DaCBF4* and *Ubi:DaCBF7* (Table 2). Cold stress generally reduces photosynthetic activity and downregulates the expression of photosynthesis-related genes (Allen and Ort, 2001; Fowler and Thomashow, 2002; Tsonev et al., 2003). In addition, photosynthesis-related genes are downregulated in *CBF*-overexpressing transgenic plants compared to wild-type plants. However, most studies did not identify the downregulated genes as upregulated genes are more important in terms of stress tolerance. An RNA-Seq study revealed that genes with diverse functions were downregulated in *AtCBF2-* and *AtCBF3-*overexpressing *Arabidopsis* lines, but photosynthesis-related genes were not over-represented among the downregulated genes (Li et al., 2017). However, in this study, the expression of photosynthesis-related genes was decreased in both transgenic rice lines; therefore, the mechanism by which *DaCBF*s regulate the expression of genes involved in photosynthesis warrants further investigation.

## Conclusion

Our results showed that heterologous expression of Antarctic hairgrass *DaCBF4* increased the tolerance of transgenic rice plants to low-temperature stress. A comparative transcriptome analysis identified common downstream targets of DaCBF4 and DaCBF7 that resulted in the same phenotype. The genes identified in this study will facilitate genetic engineering of cereal plants with enhanced cold tolerance.

## Author contributions

WTK and HL: conceived and designed the study; MYB, LHC, and AL: performed the experiments; MYB, LHC, JL, and HP: analyzed the data; MYB, LHC, WTK, and HL: discussed the results and wrote the manuscript.

### Conflict of interest statement

The authors declare that the research was conducted in the absence of any commercial or financial relationships that could be construed as a potential conflict of interest.

## Abbreviations

CBF: C-repeat binding factor
CRT: C repeat
DEG: differentially expressed gene
DRE: dehydration-responsive element
FDR: false discovery rate
RT-qPCR: Real-time reverse transcription-quantitative polymerase chain reaction
RNA-seq: RNA sequencing
FPKM: fragments per kilobase of exon model per million mapped reads
sGFP: synthetic green fluorescent protein
Ubi: ubiquitin.

## References

[B1] Agarwal P. Agarwal P. K. Joshi A. J. Sopory S. K. Reddy M. K. (2010). Overexpression of *PgDREB2A* transcription factor enhances abiotic stress tolerance and activates downstream stress-responsive genes. Mol. Biol. Rep. 37, 1125–1135. 10.1007/s11033-009-9885-8 19826914

[B2] Alberdi M. Bravo L. A. Gutiérrez A. Gidekel M. Corcuera L. J. (2002). Ecophysiology of Antarctic vascular plants. Physiol. Plant. 115, 479–486. 10.1034/j.1399-3054.2002.1150401.x 12121453

[B3] Allen D. J. Ort D. R. (2001). Impacts of chilling temperatures on photosynthesis in warm-climate plants. Trends Plant Sci. 6, 36–42. 10.1016/S1360-1385(00)01808-2 11164376

[B4] Arasan S. K. T. Park J. I. Ahmed N. U. Jung H. J. Hur Y. Kang K. K.. (2013). Characterization and expression analysis of dirigent family genes related to stresses in *Brassica*. Plant Physiol. Biochem. 67, 144–153. 10.1016/j.plaphy.2013.02.030 23562798

[B5] Artus N. N. Uemura M. Steponkus P. L. Gilmour S. J. Lin C. Thomashow M. F. (1996). Constitutive expression of the cold-regulated *Arabidopsis thaliana COR15a* gene affects both chloroplast and protoplast freezing tolerance. Proc. Natl. Acad. Sci. U.S.A. 93, 13404–13409. 10.1073/pnas.93.23.13404 11038526 PMC24106

[B6] Badawi M. Danyluk J. Boucho B. Houde M. Sarhan F. (2007). The CBF gene family in hexaploid wheat and its relationship to the phylogenetic complexity of cereal CBFs. Mol. Genet. Genomics 277, 533–554. 10.1007/s00438-006-0206-9 17285309 PMC2491707

[B7] Bao F. Du D. An Y. Yang W. Wang J. Cheng T.. (2017). Overexpression of *Prunus mume* dehydrin genes in tobacco enhances tolerance to cold and drought. Front. Plant Sci. 8:151. 10.3389/fpls.2017.00151 28224001 PMC5293821

[B8] Battaglia M. Olvera-Carrillo Y. Garciarrubio A. Campos F. Covarrubias A. A. (2008). The enigmatic LEA proteins and other hydrophilins. Plant Physiol. 148, 6–24. 10.1104/pp.108.120725 18772351 PMC2528095

[B9] Bravo L. A. Griffith M. (2005). Characterization of antifreeze activity in Antarctic plants. J. Exp. Bot. 56, 1189–1196. 10.1093/jxb/eri112 15723822

[B10] Byun M. Y. Cui L. H. Oh T. K. Jung Y. -J. Lee A. Park K. Y.. (2017). Homologous U-box E3 ubiquitin ligases OsPUB2 and OsPUB3 are involved in the positive regulation of low temperature stress response in rice (*Oryza sativa* L.). Front. Plant Sci. 8:16. 10.3389/fpls.2017.00016 28163713 PMC5247461

[B11] Byun M. Y. Kim W. T. (2014). Suppression of *OsRAD51D* results in defects in reproductive development in rice (*Oryza sativa* L.). Plant J. 79, 256–269. 10.1111/tpj.12558 24840804

[B12] Byun M. Y. Lee J. Cui L. H. Kang Y. Oh T. K. Park H.. (2015). Constitutive expression of *DaCBF7*, an Antarctic vascular plant *Deschampsia antarctica* CBF homolog, resulted in improved cold tolerance in transgenic rice plants. Plant Sci. 236, 61–74. 10.1016/j.plantsci.2015.03.020 26025521

[B13] Candat A. Paszkiewicz G. Neveu M. Gautier R. Logan D. C. Avelange-Macherel M. H.. (2014). The ubiquitous distribution of late embryogenesis abundant proteins across cell compartments in *Arabidopsis* offers tailored protection against abiotic stress. Plant Cell 26, 3148–3166. 10.1105/tpc.114.127316 25005920 PMC4145138

[B14] Chen L. Han J. Deng X. Tan S. Li L. Li L.. (2016). Expansion and stress responses of AP2/EREBP superfamily in *Brachypodium distachyon*. Sci. Rep. 6:21623. 10.1038/srep21623 26869021 PMC4751504

[B15] Claeys H. Inzé D. (2013). The agony of choice: how plants balance growth and survival under water-limiting conditions. Plant Physiol. 162, 1768–1779. 10.1104/pp.113.220921 23766368 PMC3729759

[B16] Cui M. Zhang W. Zhang Q. Xu Z. Zhu Z. Duan F.. (2011). Induced over-expression of the transcription factor *OsDREB2A* improves drought tolerance in rice. Plant Physiol. Biochem. 49, 1384–1391. 10.1016/j.plaphy.2011.09.012 22078375

[B17] Drira M. Saibi W. Brini F. Gargouri A. Masmoudi K. Hanin M. (2013). The K-segments of the wheat dehydrin DHN-5 are essential for the protection of lactate dehydrogenase and β-glucosidase activities *in vitro*. Mol. Biotechnol. 54, 643–650. 10.1007/s12033-012-9606-8 23054631

[B18] Dubouzet J. G. Sakuma Y. Ito Y. Kasuga M. Dubouzet E. G. Miura S.. (2003). *OsDREB* genes in rice, *Oryza sativa* L., encode transcription activators that function in drought-, high-salt-and cold-responsive gene expression. Plant J. 33, 751–763. 10.1046/j.1365-313X.2003.01661.x 12609047

[B19] Fowler S. Thomashow M. F. (2002). *Arabidopsis* transcriptome profiling indicates that multiple regulatory pathways are activated during cold acclimation in addition to the CBF cold response pathway. Plant Cell 14, 1675–1690. 10.1105/tpc.003483 12172015 PMC151458

[B20] Gao S. Q. Chen M. Xia L. Q. Xiu H. J. Xu Z. S. Li L. C.. (2009). A cotton (*Gossypium hirsutum*) DRE-binding transcription factor gene, *GhDREB*, confers enhanced tolerance to drought, high salt, and freezing stresses in transgenic wheat. Plant Cell Rep. 28, 301–311. 10.1007/s00299-008-0623-9 19005655

[B21] Giełwanowska I. Szczuka E. Bednara J. Górecki R. (2005). Anatomical features and ultrastructure of *Deschampsia antarctica* (Poaceae) leaves from different growing habitats. Ann. Bot. 96, 1109–1119. 10.1093/aob/mci262 16157630 PMC4247099

[B22] Gilmour S. J. Fowler S. G. Thomashow M. F. (2004). *Arabidopsis* transcriptional activators CBF1, CBF2, and CBF3 have matching functional activities. Plant Mol. Biol. 54, 767–781. 10.1023/B:PLAN.0000040902.06881.d4 15356394

[B23] Hillwig M. S. (2009). Regulation, Function, and Evolution of T2 RNases. Graduate theses and dissertations. Available online at: http://lib.dr.iastate.edu/etd/11095

[B24] Hundertmark M. Hincha D. K. (2008). LEA (late embryogenesis abundant) proteins and their encoding genes in *Arabidopsis thaliana*. BMC Genomics 9:118. 10.1186/1471-2164-9-118 18318901 PMC2292704

[B25] Ito Y. Katsura K. Maruyama K. Taji T. Kobayashi M. Seki M.. (2006). Functional analysis of rice DREB1/CBF-type transcription factors involved in cold-responsive gene expression in transgenic rice. Plant Cell Physiol. 47, 141–153. 10.1093/pcp/pci230 16284406

[B26] Jaglo-Ottosen K. R. Gilmour S. J. Zarka D. G. Schabenberger O. Thomashow M. F. (1998). *Arabidopsis CBF1* overexpression induces *COR* genes and enhances freezing tolerance. Science 280, 104–106. 10.1126/science.280.5360.104 9525853

[B27] Jeknić Z. Pillman K. A. Dhillon T. Skinner J. S. Veisz O. Cuesta-Marcos A.. (2014). *Hv-CBF2A* overexpression in barley accelerates *COR* gene transcript accumulation and acquisition of freezing tolerance during cold acclimation. Plant Mol. Biol. 84, 67–82. 10.1007/s11103-013-0119-z 23949371

[B28] John U. P. Polotnianka R. M. Sivakumaran K. A. Chew O. Mackin L. Kuiper M. J.. (2009). Ice recrystallization inhibition proteins (IRIPs) and freeze tolerance in the cryophilic Antarctic hair grass *Deschampsia antarctica* E. Desv. Plant Cell Environ. 32, 336–348. 10.1111/j.1365-3040.2009.01925.x 19143989

[B29] Kidrič M. Kos J. Sabotič J. (2014). Proteases and their endogenous inhibitors in the plant response to abiotic stress. Bot. Serb. 38, 139–158.

[B30] Koag M. C. Fenton R. D. Wilkens S. Close T. J. (2003). The binding of maize DHN1 to lipid vesicles. Gain of structure and lipid specificity. Plant Physiol. 131, 309–316. 10.1104/pp.011171 12529538 PMC166810

[B31] Kosová K. Vítámvás P. Prášil I. T. (2014). Wheat and barley dehydrins under cold, drought, and salinity - what can LEA-II proteins tell us about plant stress response? Front. Plant Sci. 5:343. 10.3389/fpls.2014.00343 25071816 PMC4089117

[B32] Kovalchuk N. Jia W. Eini O. Morran S. Pyvovarenko T. Fletcher S.. (2013). Optimization of *TaDREB3* gene expression in transgenic barley using cold-inducible promoters. Plant Biotechnol. J. 11, 659–670. 10.1111/pbi.12056 23495849

[B33] Kumar M. Lee S. C. Kim J. Y. Kim S. J. Aye S. S. Kim S.-R. (2014). Over-expression of dehydrin gene, *OsDhn1*, improves drought and salt stress tolerance through scavenging of reactive oxygen species in rice (*Oryza sativa* L.). J. Plant Biol. 57, 383–393. 10.1007/s12374-014-0487-1

[B34] Kumar S. Stecher G. Tamura K. (2016). MEGA7: Molecular Evolutionary Genetics Analysis version 7.0 for bigger datasets. Mol. Biol. Evol. 33, 1870–1874. 10.1093/molbev/msw054 27004904 PMC8210823

[B35] Kume S. Kobayashi F. Ishibashi M. Ohno R. Nakamura C. Takumi S. (2005). Differential and coordinated expression of *Cbf* and *Cor*/*Lea* genes during long-term cold acclimation in two wheat cultivars showing distinct levels of freezing tolerance. Genes Genet. Syst. 80, 185–197. 10.1266/ggs.80.185 16172531

[B36] Kuwabara C. Takezawa D. Shimada T. Hamada T. Fujikawa S. Arakawa K. (2002). Abscisic acid-and cold-induced thaumatin-like protein in winter wheat has an antifungal activity against snow mould, *Microdochium nivale*. Physiol. Plant. 115, 101–110. 10.1034/j.1399-3054.2002.1150112.x 12010473

[B37] Lata C. Prasad M. (2011). Role of DREBs in regulation of abiotic stress responses in plants. J. Exp. Bot. 62, 4731–4748. 10.1093/jxb/err210 21737415

[B38] Lee H. Kim J. H. Park M. Kim I. C. Yim J. H. Lee H. K. (2010). Reference genes validation for qPCR normalization in *Deschampsia antarctica* during abiotic stresses. Antarct. Sci. 22, 477–484. 10.1017/S0954102010000428

[B39] Lee J. Noh E. K. Choi H. S. Shin S. C. Park H. Lee H. (2013a). Transcriptome sequencing of the Antarctic vascular plant *Deschampsia antarctica* Desv. under abiotic stress. Planta 237, 823–836. 10.1007/s00425-012-1797-5 23135329

[B40] Lee J. Noh E. K. Park H. Lee H. (2013b). Transcription factor profile analysis of the Antarctic vascular plant *Deschampsia antarctica* Desv. (Poaceae). Genes Genom. 35, 575–586. 10.1007/s13258-013-0106-4

[B41] Li A. Zhou M. Wei D. Chen H. You C. Lin J. (2017). Transcriptome profiling reveals the negative regulation of multiple plant hormone signaling pathways elicited by overexpression of C-repeat binding factors. Front. Plant Sci. 8:1647. 10.3389/fpls.2017.01647 28983312 PMC5613223

[B42] Liu Y. Song Q. Li D. Yang X. Li D. (2017). Multifunctional roles of plant dehydrins in response to environmental stresses. Front. Plant Sci. 8:1018. 10.3389/fpls.2017.01018 28649262 PMC5465263

[B43] Misra R. C. Kamthan M. Kumar S. Ghosh S. (2016). A thaumatin-like protein of *Ocimum basilicum* confers tolerance to fungal pathogen and abiotic stress in transgenic Arabidopsis. Sci. Rep. 6:25340. 10.1038/srep25340 27150014 PMC4858651

[B44] Molina A. Diaz I. Carbonero P. García-Olmedo F. Vasil I. K. (1996). Two cold-inducible genes encoding lipid transfer protein LTP4 from barley show differential responses to bacterial pathogens. Mol. Gen. Genet. 252, 162–168. 10.1007/BF02173216 8804389

[B45] Morran S. Eini O. Pyvovarenko T. Parent B. Singh R. Ismagul A.. (2011). Improvement of stress tolerance of wheat and barley by modulation of expression of DREB/CBF factors. Plant Biotechnol. J. 9, 230–249. 10.1111/j.1467-7652.2010.00547.x 20642740

[B46] Mortazavi A. Williams B. A. McCue K. Schaeffer L. Wold B. (2008). Mapping and quantifying mammalian transcriptomes by RNA-Seq. Nat. Methods 5, 621–628. 10.1038/nmeth.1226 18516045 PMC13303166

[B47] Oh S. J. Kwon C. W. Choi D. W. Song S. I. Kim J. K. (2007). Expression of barley *HvCBF4* enhances tolerance to abiotic stress in transgenic rice. Plant Biotechnol. J. 5, 646–656. 10.1111/j.1467-7652.2007.00272.x 17614953

[B48] Oh S. J. Song S. I. Kim Y. S. Jang H. J. Kim S. Y. Kim M.. (2005). Arabidopsis CBF3/DREB1A and ABF3 in transgenic rice increased tolerance to abiotic stress without stunting growth. Plant Physiol. 138, 341–351. 10.1104/pp.104.059147 15834008 PMC1104188

[B49] Paniagua C. Bilkova A. Jackson P. Dabravolski S. Riber W. Didi V.. (2017). Dirigent proteins in plants: modulating cell wall metabolism during abiotic and biotic stress exposure. J. Exp. Bot. 18, 3287–3301. 10.1093/jxb/erx141 28472349

[B50] Pastorczyk M. Giełwanowska I. Lahuta L. B. (2014). Changes in soluble carbohydrates in polar Caryophyllaceae and Poaceae plants in response to chilling. Acta Physiol. Plant 36, 1771–1780. 10.1007/s11738-014-1551-7

[B51] Rawlings N. D. (2010). Peptidase inhibitors in the MEROPS database. Biochimie 92, 1463–1483. 10.1016/j.biochi.2010.04.013 20430064

[B52] Sáez P. L. Bravo L. A. Cavieres L. A. Vallejos V. Sanhueza C. Font-Carrascosa M.. (2017). Photosynthetic limitations in two Antarctic vascular plants: importance of leaf anatomical traits and Rubisco kinetic parameters. J. Exp. Bot. 68, 2871–2883. 10.1093/jxb/erx148 28830100 PMC5854023

[B53] Sasaki K. Christov N. K. Tsuda S. Imai R. (2014). Identification of a novel LEA protein involved in freezing tolerance in wheat. Plant Cell Physiol. 55, 136–147. 10.1093/pcp/pct164 24265272

[B54] Skinner J. S. von Zitzewitz J. Szucs P. Marquez-Cedillo L. Filichkin T. Amundsen K.. (2005). Structural, functional, and phylogenetic characterization of a large *CBF* gene family in barley. Plant Mol. Biol. 59, 533–551. 10.1007/s11103-005-2498-2 16244905

[B55] Soltész A. Smedley M. Vashegyi I. Galiba G. Harwood W. Vágújfalvi A. (2013). Transgenic barley lines prove the involvement of *TaCBF14* and *TaCBF15* in the cold acclimation process and in frost tolerance. J. Exp. Bot. 64, 1849–1862. 10.1093/jxb/ert050 23567863 PMC3638819

[B56] Sror H. A. Tischendorf G. Sieg F. Schmitt J. M. Hincha D. K. (2003). Cryoprotectin protects thylakoids during a freeze–thaw cycle by a mechanism involving stable membrane binding. Cryobiology 47, 191–203. 10.1016/j.cryobiol.2003.09.005 14697731

[B57] Stockinger E. J. (2009). Winter hardiness and the *CBF* genes in the Triticeae, in Plant Cold Hardiness: From the Laboratory to the Field, eds Gusta L. V. Tanino K. K. Wisniewski M. E. (Cambridge: CAB International), 119–130.

[B58] Thomashow M. F. (2010). Molecular basis of plant cold acclimation: insights gained from studying the CBF cold response pathway. Plant Physiol. 154, 571–577. 10.1104/pp.110.161794 20921187 PMC2948992

[B59] Tian T. Liu Y. Yan H. You Q. Yi X. Du Z.. (2017). agriGO v2.0: a GO analysis toolkit for the agricultural community, 2017 update. Nucleic Acids Res. 45, W122–W129. 10.1093/nar/gkx382 28472432 PMC5793732

[B60] Tondelli A. Francia E. Barabaschi D. Pasquariello M. Pecchioni N. (2011). Inside the *CBF* locus in *Poaceae*. Plant Sci. 180, 39–45. 10.1016/j.plantsci.2010.08.012 21421345

[B61] Tsonev T. Velikova V. Georgieva K. Hyde P. F. Jones H. G. (2003). Low temperature enhances photosynthetic downregulation in French bean (*Phaseolus vulgaris* L.) plants. Ann. Bot. 91, 343–352. 10.1093/aob/mcg020 12547687 PMC4244959

[B62] Vágújfalvi A. Aprile A. Miller A. Dubcovsky J. Delugu G. Galiba G.. (2005). The expression of several *Cbf* genes at the *Fr-A2* locus is linked to frost resistance in wheat. Mol. Genet. Genomics 274, 506–514. 10.1007/s00438-005-0047-y 16200412

[B63] Wang X. S. Zhu H. B. Jin G. L. Liu H. L. Wu W. R. Zhu J. (2007). Genome-scale identification and analysis of *LEA* genes in rice (*Oryza sativa* L.). Plant Sci. 172, 414–420. 10.1016/j.plantsci.2006.10.004

[B64] Wei T. Deng K. Zhang Q. Gao Y. Liu Y. Yang M.. (2017). Modulating *AtDREB1C* expression improves drought tolerance in *Salvia miltiorrhiza*. Front. Plant Sci. 8:52. 10.3389/fpls.2017.00052 28174590 PMC5259653

[B65] Wu G. Robertson A. J. Liu X. Zheng P. Wilen R. W. Nesbitt N. T.. (2004). A lipid transfer protein gene *BG-14* is differentially regulated by abiotic stress, ABA, anisomycin, and sphingosine in bromegrass (*Bromus inermis*). J. Plant Physiol. 161, 449–458. 10.1078/0176-1617-01259 15128032

[B66] Wu R. Wang L. Wang Z. Shang H. Liu X. Zhu Y. (2009). Cloning and expression analysis of a dirigent protein gene from the resurrection plant *Boea hygrometrica*. Prog. Nat. Sci. 19, 347–352. 10.1016/j.pnsc.2008.07.010

[B67] Xu M. Li L. Fan Y. Wan J. Wang L. (2011). ZmCBF3 overexpression improves tolerance to abiotic stress in transgenic rice (*Oryza sativa*) without yield penalty. Plant Cell Rep. 30, 1949–1957. 10.1007/s00299-011-1103-1 21811828

[B68] Zheng J. Wang Y. He Y. Zhou J. Li Y. Liu Q.. (2014). Overexpression of an S-like ribonuclease gene, *OsRNS4*, confers enhanced tolerance to high salinity and hyposensitivity to phytochrome-mediated light signals in rice. Plant Sci. 214, 99–105. 10.1016/j.plantsci.2013.10.003 24268167

