# Supplementary material for: Identification of Rice Genes Associated With Enhanced Cold Tolerance by Comparative Transcriptome Analysis With Two Transgenic Rice Plants Overexpressing DaCBF4 or DaCBF7, Isolated From Antarctic Flowering Plant Deschampsia antarctica
Source: Front Plant Sci. 2018 May 3;9:601. doi: 10.3389/fpls.2018.00601 (PMC5943562; doi:10.3389/fpls.2018.00601)
Supplement: Supplementary file 7 [file Image_1.PDF]

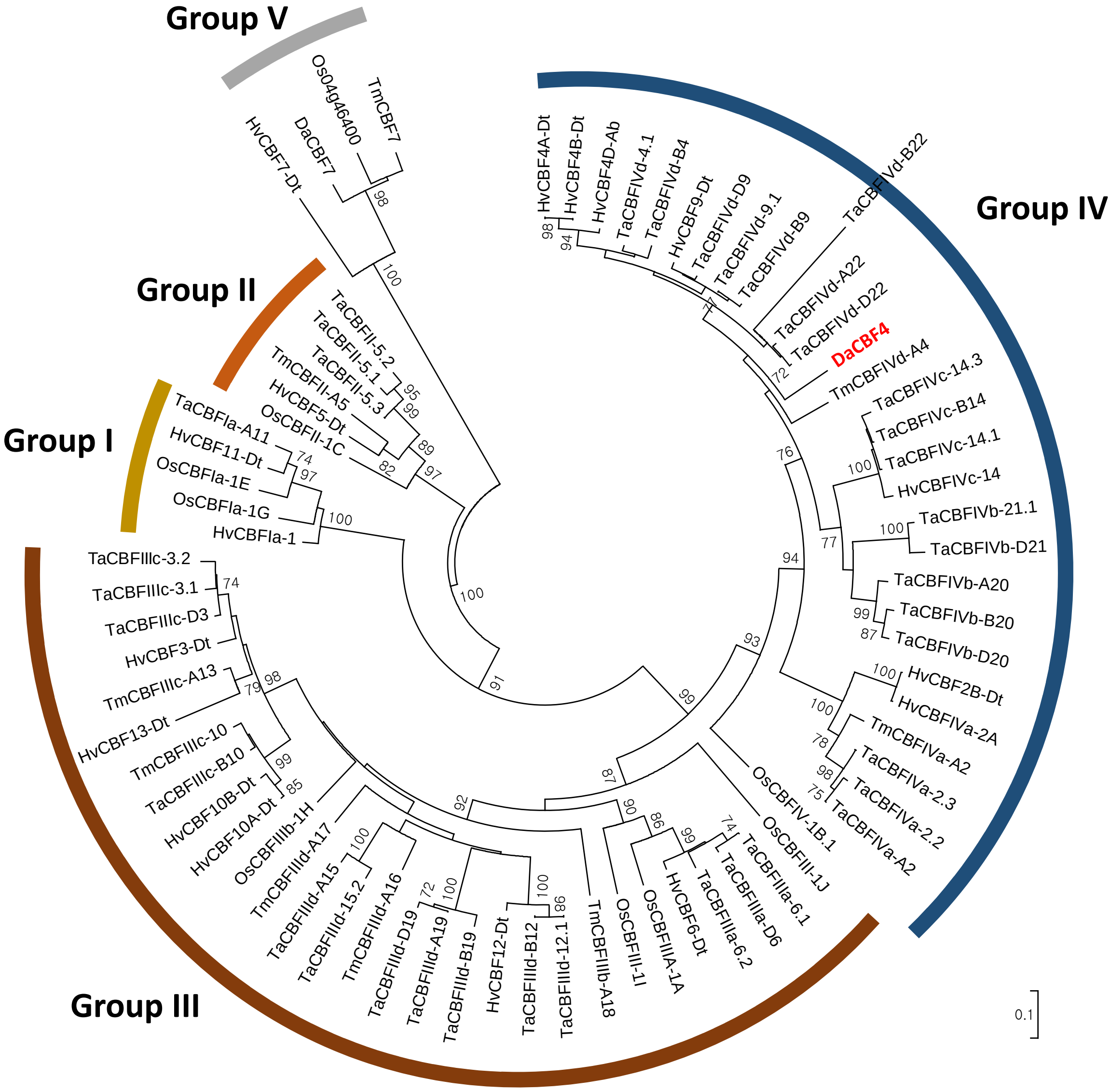

Supplementary Figure 1. Phylogenetic analysis of DaCBF4. DaCBF4 is represented in red letters and groups are labeled based on the work of Badawi *et al.* (2007) and Byun et al. (2015). The monocot CBF members are divided into five different groups. The evolutionary history was inferred using the neighbor joining method using MEGA7. The bootstrap values (> 70) were presented near the corresponding branch. Accession numbers of CBF homologs from five monocot species including Antarctic hairgrass (*Deschampsia antarctica*), barley (*Hordeum vulgare*), rice (*Oryza sativa*), hexaploid wheat (*Triticum aestivum*), and einkorn wheat (*Triticum monococcum*) are presented in Supplementary Table 1.

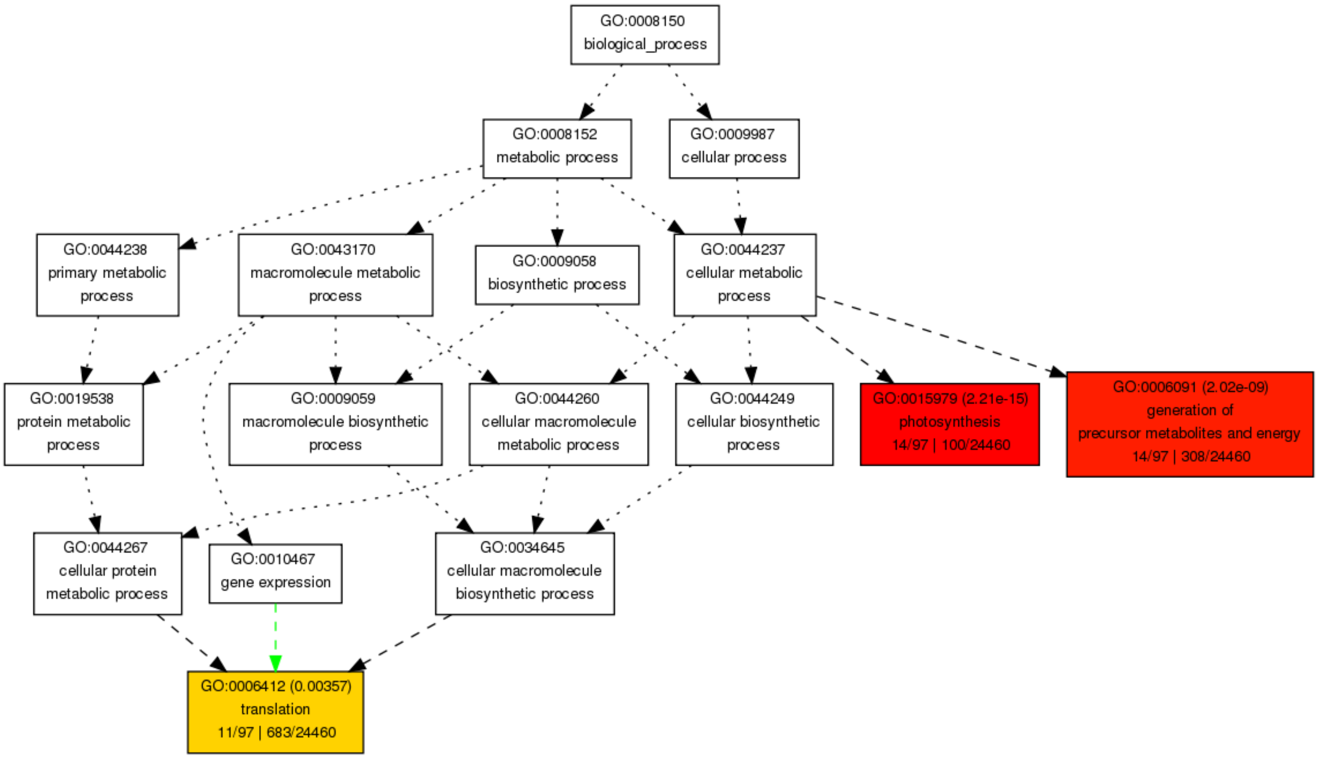

Supplementary Figure 2. Functional GO classification of DEGs by singular enrichment analysis of plant GO slim. Only GO terms of DEGs with significance calculated by Yekutieli multi-test adjustment method (corrected *p*-value of FDR < 0.05) are presented with different colors when comparing with those of rice whole genome. The box colors indicates levels of statistical significance with yellow = 0.05 and red = e-09.
